# Strategies to access the [5-8] bicyclic core encountered in the sesquiterpene, diterpene and sesterterpene series

**DOI:** 10.3762/bjoc.19.23

**Published:** 2023-03-03

**Authors:** Cécile Alleman, Charlène Gadais, Laurent Legentil, François-Hugues Porée

**Affiliations:** 1 Université Rennes, Faculté de Pharmacie, CNRS ISCR UMR 6226, F-35000 Rennes, France; 2 Université Rennes, Ecole Nationale Supérieure de Chimie de Rennes, CNRS, ISCR – UMR 6226, F-35000 Rennes, France

**Keywords:** 5-8 bicycle, cyclization strategies, terpenes

## Abstract

Terpene compounds probably represent the most diversified class of secondary metabolites. Some classes of terpenes, mainly diterpenes (C20) and sesterterpenes (C25) and to a lesser extent sesquiterpenes (C15), share a common bicyclo[3.6.0]undecane core which is characterized by the presence of a cyclooctane ring fused to a cyclopentane ring, i.e., a [5-8] bicyclic ring system. This review focuses on the different strategies elaborated to construct this [5-8] bicyclic ring system and their application in the total synthesis of terpenes over the last two decades. The overall approaches involve the construction of the 8-membered ring from an appropriate cyclopentane precursor. The proposed strategies include metathesis, Nozaki–Hiyama–Kishi (NHK) cyclization, Pd-mediated cyclization, radical cyclization, Pauson–Khand reaction, Lewis acid-promoted cyclization, rearrangement, cycloaddition and biocatalysis.

## Introduction

**Figure 1:**
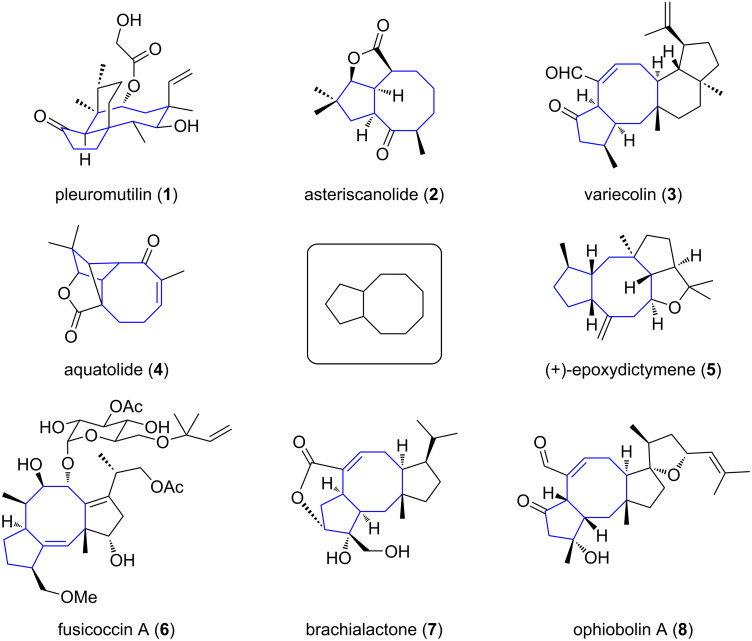
Examples of terpenes containing a bicyclo[3.6.0]undecane motif.

Terpene compounds represent the largest and most diversified class of secondary metabolites. They are present in all organisms and their structure can vary from simple terpenes (C10 skeleton) to polymers (example of rubber) thanks to iterative addition of geranyl (C10) or farnesyl (C15) building blocks derived from isoprene as starting unit and further structure rearrangement and functionalization [1]. This ubiquitous distribution highlights their pivotal role in living systems such as cell wall structural agent or ecological mediator [2]. In several cases, terpenes possess a complex polycyclic framework which challenged the chemistry community over the years to develop efficient approaches and methodologies to access them. Indeed, some classes of terpenes, mainly diterpenes (C20) and sesterterpenes (C25) and to a lesser extent sesquiterpenes (C15), share a common bicyclo[3.6.0]undecane core which is characterized by the presence of a cyclooctane ring fused to a cyclopentane ring i.e., a [5-8] bicyclic ring system (Figure 1). In most cases, additional rings are present and original strategies have been considered to construct this bicyclic system.

This review focuses on the different strategies elaborated to build this [5-8] bicyclic ring system and their applications in the total synthesis of terpenes over the last two decades [3]. We are not providing here a comprehensive collection of the literature. When appropriate, the reader will be referred to specific reviews on a particular class of terpenes. Rather, we try to present the portfolio of available methods used nowadays to build this motif.

Accordingly, in most cases the overall approaches involve construction of the 8-membered ring from an appropriate cyclopentane precursor. The proposed strategies include metathesis, Nozaki–Hiyama–Kishi (NHK) cyclization, Pd-mediated cyclization, radical cyclization (including SmI_2_), Pauson–Khand reaction, Lewis acid-promoted cyclization, rearrangement, cycloaddition, and biocatalysis. In particular, the purpose will focus on the following criteria: position/stage of the key cyclooctane ring formation in the synthesis plan, the selectivity, and the opportunity for late-stage functionalization.

## Review

### 1 Metathesis: ring-closing metathesis and related methods

**Figure 2:**
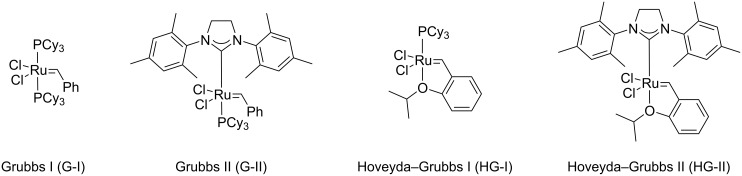
Commercially available first and second generation Grubbs and Hoveyda–Grubbs catalysts.

The metathesis reaction, first discovered by serendipity in the 1950s, has turned into one of the most used and powerful reactions in organic synthesis, and allows the formation of functionalized double or triple bonds. Over the time, this reaction drew more and more scientists’ attention, with increasing numbers of publications on this topic [4–6]. Proof of this success, Chauvin, Schrock and Grubbs were awarded the Nobel Prize in 2005 for their improvements in metathesis reactions [5–6]. Key to success of the metathesis reaction arises from its broad spectrum of application, its functional groups tolerance, its versatility, and its mild reaction conditions [4–9]. Metathesis reactions take place by the means of a metallic catalyst. Firstly, olefin metathesis was achieved with an air-sensitive tungsten complex [8]. An important focus on air-stable catalyst design was undertaken and contributed to the popularization of the reaction. Thus, Grubbs catalysts of 1st and 2nd generation (G-I and G-II, respectively) as well as more recent Hoveyda–Grubbs 1st or 2nd generation (HG-I and HG-II, respectively) (Figure 2) are now commercially available. Today, the design of new efficient catalysts is at the heart of research [4–7,9–12].

The synthesis of five- and six-membered rings can be readily achieved through ring-closing metathesis (RCM), but the synthesis of eight-membered rings has been carried out later [13–14]. In this part, we will focus on the use of RCM to achieve total syntheses of natural products or to access the carbon skeleton of natural products, and more particularly to construct the cyclooctane ring that is part in these motifs.

#### 1.1 Ring-closing metathesis (RCM)

Among all metathesis reactions available in the chemist’s toolbox, ring-closing metathesis (RCM) is nowadays one of the most popular and has been used several times as a key-step to synthesize the cyclooctanoid ring that is part of the sesterterpenoid family.

**1.1.1 Syntheses of fusicoccans and ophiobolins:** Fusicoccanes and ophiobolins represent an important class of natural diterpenes, whose principal members are fusicoccin A (**6**) and ophiobolin A (**8**), respectively (Figure 1). Both families share the same core structure bearing a fused [5-8-5] tricyclic skeleton and they vary within their decorative functional groups.

**Figure 3:**
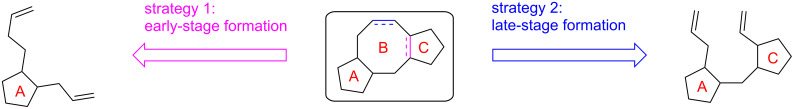
Examples of strategies to access the fusicoccan and ophiobolin tricyclic core structure by RCM.

Over the years, many different syntheses to access compounds from the fusicoccan and ophiobolin family have been reported, with two strategies standing out. The first one (strategy 1, Figure 3) consists in the successive formation of the eight-membered ring B followed by construction of the five-membered ring C: i.e., the cyclooctene ring is built first from a suitably substituted cyclopentane substrate, which then serves as a precursor for the cyclopentane ring C formation. In the second strategy (strategy 2, Figure 3), both cyclopentane rings A and C are formed first, and the eight-membered ring B is introduced at a late stage.

**Scheme 1:**
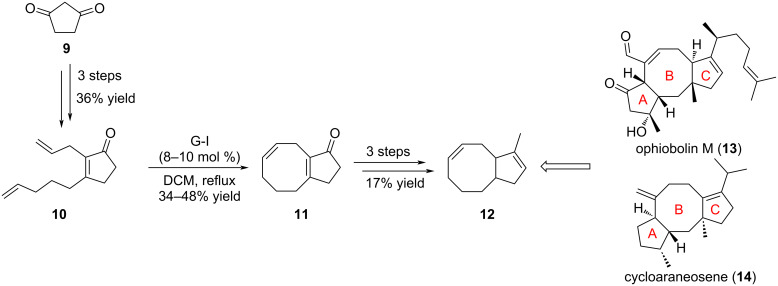
Synthesis of bicyclic core structure **12** of ophiobolin M (**13**) and cycloaraneosene (**14**).

**1.1.1.1 Strategy 1:** Successive introduction of rings B and C starting from cyclopentene via the [5-8] bicyclic structure to the final [5-8-5] tricyclic core:

The synthesis of the core bicyclic structure **12** of ophiobolin M (**13**) isolated from the fungus *Cochliobolus heterostrophus* and cycloaraneosene (**14**) firstly isolated in 1975 from the fungus *Sordaria araneosa*, was undertaken by Williams et al*.* in 2002 [15]. Compound **14** shares the common [5-8-5] tricyclic framework emblematic of the fusicoccan series, albeit cycloaraneosene (**14**) does not have any heteroatom (Scheme 1) [16]. Focusing on the B–C bicyclic fragment, they used diene **10**, readily available from cyclopentane-1,3-dione (**9**), as the precursor for installing the eight-membered ring by RCM (Scheme 1). Optimization of the reaction parameters (temperature, solvent, concentration, and catalyst amount) was carried out with G-I catalyst. Indeed, the authors noticed that high temperatures improved the reaction conversion, and dichloromethane seemed to furnish clean reaction and easy work-up. Thus, the final conditions were determined as 8–10 mol % of G-I catalyst loading in refluxing dichloromethane, albeit C5-C8 bicycle **11** was formed in modest yields and incomplete conversion. Interestingly, use of G-II catalyst, known to give better results, did not improve the yield nor the bicycle-to-monocycle ratio.

**Scheme 2:**
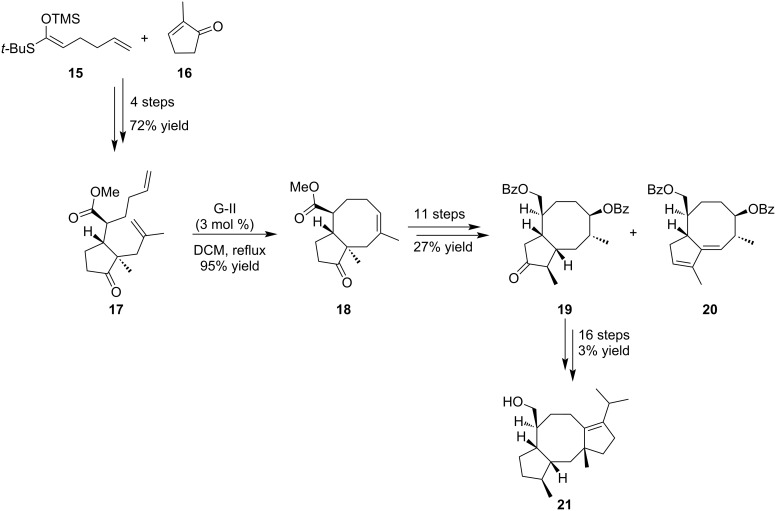
Synthesis of the core structure **21** of ophiobolins and fusicoccanes.

In 2005, Michalak et al. examined the synthesis of the dicyclopenta[*a,d*]cyclooctane structure present in ophiobolins and fusicoccanes by the means of a ring-closing metathesis reaction [17]. The metathesis substrate **17** was readily prepared in four steps from accessible methylcyclopentenone **16** (Scheme 2). The sequence included a Mukaiyama–Michael reaction with silyl enol **15** followed by a Tsuji alkylation. With diene **17** in hands, the RCM reaction was performed by addition of G-II catalyst and furnished the expected C5-C8 bicyclic framework **18** in 95% yield [17–18]. This key intermediate was then converted into ketone **19** in 11 steps leading to the desired dicyclopenta[*a,d*]cyclooctane structure **21** [19].

**Scheme 3:**
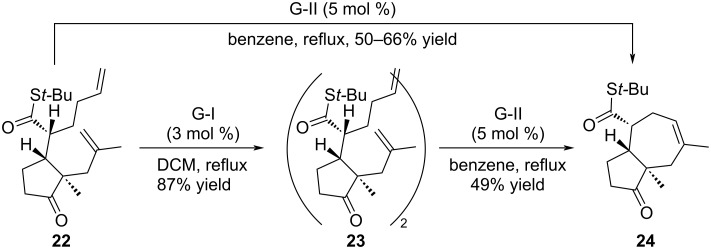
Ring-closing metathesis attempts starting from thioester **22**.

As explained by the authors, the RCM reaction was not as easy as expected and extensive work was necessary to accomplished the construction of this eight-membered ring. Indeed, in the initial strategy, thioester **22** was the first substrate subjected to the cyclization. In the presence of G-I catalyst, the reaction delivered the dimeric compound **23** whereas [5-7] bicycle **24** was formed in the presence of the G-II catalyst (Scheme 3). The formation of compound **24** was rationalized by an isomerization of the double bond prior to the cyclization step.

**Scheme 4:**
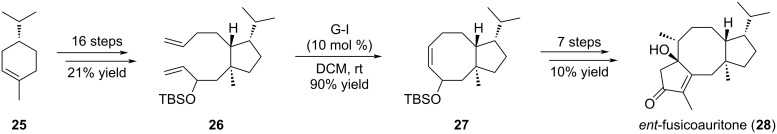
Total synthesis of *ent*-fusicoauritone (**28**).

Isolated from the Ecuadorian liverwort *Anastrophyllum auritum* in 1994, *ent*-fusicoauritone (**28**) is a diterpene presenting a fusicoccan skeleton with a β-hydroxyketone on the A-ring and 5 stereogenic centers [20]. Its total synthesis was explored by Srikrishna and Nagaraju in 2012 using a linear strategy (Scheme 4) [20]. Thus, menthene **25** was converted in 16 steps into cyclopentane **26** presenting two unsaturated lateral chains. This compound was then treated with G-I catalyst to give the corresponding [5-8] cyclooctene **27** in very high yield. The final stages of the synthesis corresponded to the elaboration of the second cyclopentenone ring allowing access to *ent*-fusicoauritone (**28**) in 24 steps.

**Figure 4:**
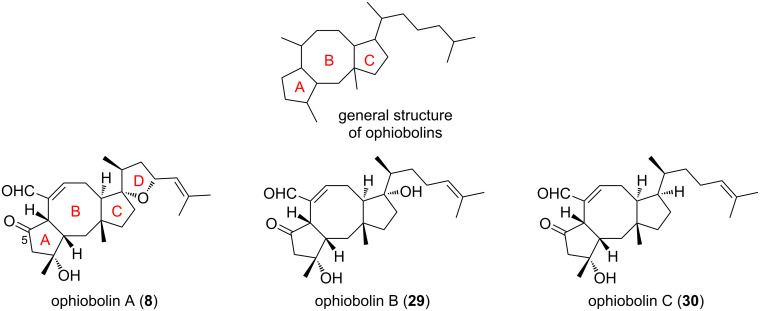
General structure of ophiobolins and congeners.

**1.1.1.2 Strategy 2:** Late-stage formation of the 8-membered ring:

Ophiobolins belong to the family of sesterterpenoids which displays around 50 members [21]. All these molecules share a similar [5-8-5] tricyclic structure (A-B-C ring system), with different decorating groups. The first congener to be isolated was ophiobolin A (**8**), which is present in the fungus *Ophiobolus miyabeanus* [22]. Other fungal species were found to produce ophiobolin A (**8**), and many other ophiobolin congeners such as ophiobolin B (**29**), ophiobolin C (**30**), and ophiobolin M (**13**) were also isolated (Figure 4) [23].

This natural products family is of high interest because of its broad spectrum of biological activities ranging from anti-infectious to anticancer properties [21,24–25]. Indeed, access to the ophiobolin structure is crucial to study their biological activities in detail. Many synthetic approaches have emerged for the synthesis of ophiobolins and total syntheses of some congeners have also been reported [21,24].

**Scheme 5:**
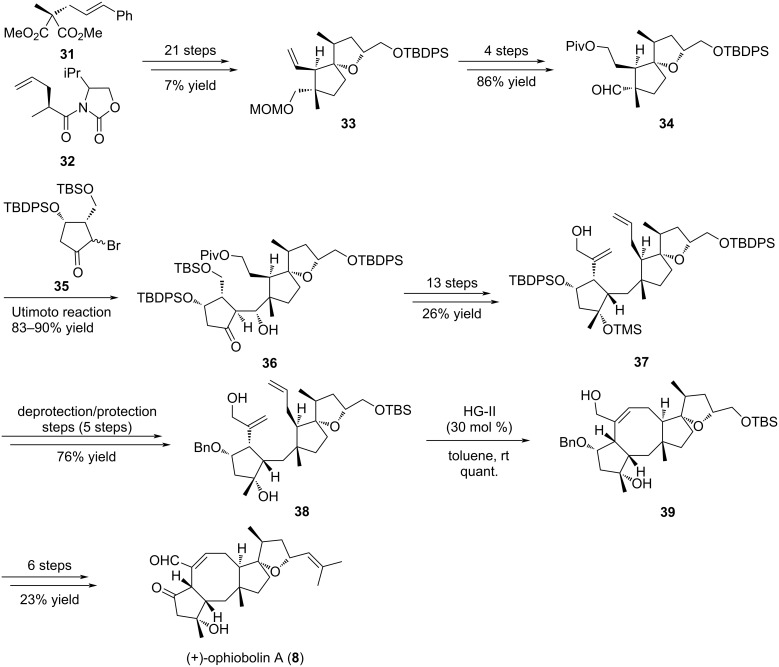
Total synthesis of (+)-ophiobolin A (**8**).

**Scheme 6:**
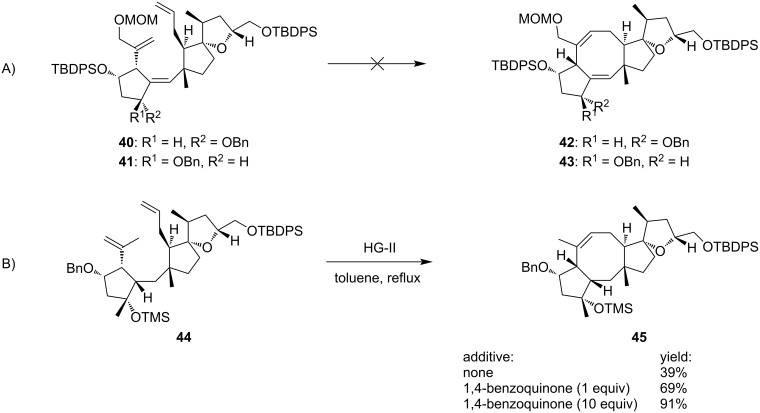
Investigation of RCM for the synthesis of ophiobolin A (**8**). Path A) RCM with TBDPS-protected alcohol. Path B) RCM with benzyl-protected alcohol.

Nakada and co-workers reported in 2011 the first enantioselective total synthesis of (+)-ophiobolin A (**8**), involving a RCM approach to form the central eight-membered ring [24]. In this way, their total synthesis involved the enantioselective preparation of the C-D spiro bicyclic ring system **33** in 21 steps from diester **31** and oxazolidinone **32** (Scheme 5) [26]. Subsequently, compound **33** was converted in four steps into aldehyde **34** which was engaged in a coupling reaction with bromoketone **35** according to Utimoto conditions to furnish the A-C-D adduct **36** as a single stereoisomer in high yield. Of note, the Utimoto reaction has never been used in the synthesis of natural products before this report, and no β-elimination of the silyloxy group was observed, although this often occurs in such systems [26]. The installation of the two alkenes in **37** required 13 additional steps, and further protecting group manipulations were necessary to give compound **38** as a precursor for the late-stage RCM cyclization. This ring formation was very challenging and necessitated extended optimization. Indeed, during the course of the RCM a dramatic effect of the OH-protecting group on the cyclopentane unit was observed. The presence of a TBDPS substituent in compound **40** was assumed unfavorable, since this bulky residue generates a steric hindrance precluding the cyclization (Scheme 6, path A) [27]. Its replacement by a sterically less demanding benzyl protecting group (compound **44**) allowed the reaction to occur (Scheme 6, path B). It also appeared that the RCM depended on the substrate core structure and the presence of a protected allylic alcohol prevented the cyclization to take place. Taking these observations into account, the tricyclic [5-8-5] ring system **45** was obtained using the HG-II catalyst in refluxing toluene. Further functionalization steps finally furnished (+)-ophiobolin A (**8**).

**Scheme 7:**
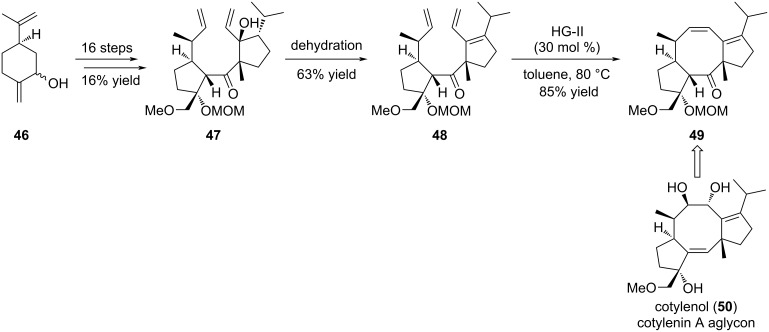
Synthesis of the core structure of cotylenin A aglycon, cotylenol (**50**).

In the fusicoccanes family, cotylenol (**50**), the aglycon of cotylenin A (**131**) (see section 3.1), is a fungal metabolite which displays various interesting biological activities [28]. For example, it expresses a moderate cytotoxicity against human myeloid leukemia cells, and stabilizes the 14-3-3 – TASK3 protein–protein interaction [29–30]. Sugita et al. investigated the synthesis of the core structure of cotylenol (**50**) first through an RCM approach on the advanced intermediate **47** (Scheme 7) [31]. Despite many attempts, the authors could not obtain the desired tricyclic compound possessing the required eight-membered ring, but instead they recovered the starting material **47**, together with a dimeric product or an eleven-membered ring resulting from a RCM and a retro-aldol reaction. Assuming that the allylic alcohol may induce a steric hindrance, alcohol **47** was converted into triene **48** upon dehydration, and further engaged in the RCM reaction. In this case, the use of HG-II catalyst proved to be the best choice to achieve cyclooctene ring formation giving rise to intermediate **49**, providing entries for further functionalization and access to cotylenin A aglycon **50**.

**Scheme 8:**
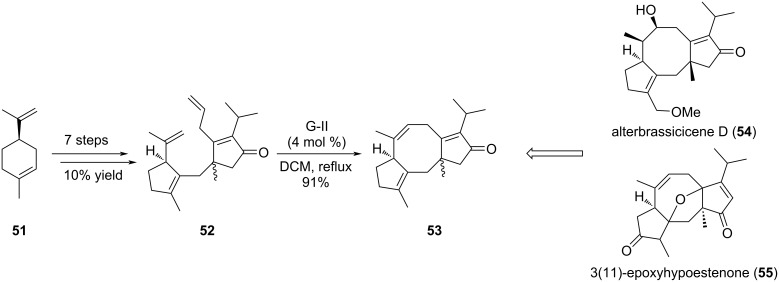
Synthesis of tricyclic core structure of fusicoccans.

Other examples of fusicoccan derivatives are represented by alterbrassicicene D (**54**) and 3(11)-epoxyhypoestenone (**55**), recently isolated from *Hypoestes verticillaris* [32]. Structurally, alterbrassicicene D (**54**) comprises an α,β-unsaturated ketone C-ring, and a single hydroxy group on the central eight-membered ring and 3(11)-epoxyhypoestenone (**55**) shows a surprising oxa-bridge between the A and C rings, an α,β-unsaturated ketone on ring C, and an *endo*-alkene into the cyclooctene ring. Recently, Chen et al. reported the synthesis of the tricyclic core structure of the fusicoccane skeleton **53** using a RCM approach (Scheme 8) [33]. The key RCM reaction was envisioned on precursor **52** corresponding to the A-C rings of the tricyclic backbone. In this case, G-II catalyst was used and the expected product **53** was obtained in high yields. Interestingly, the tricyclic intermediate **53** could be obtained in only 8 steps and at a large scale. Validation of this strategy allowed the authors to extend this approach to the total synthesis of alterbrassicicene D (**54**) and 3(11)-epoxyhypoestenone (**55**).

**Scheme 9:**
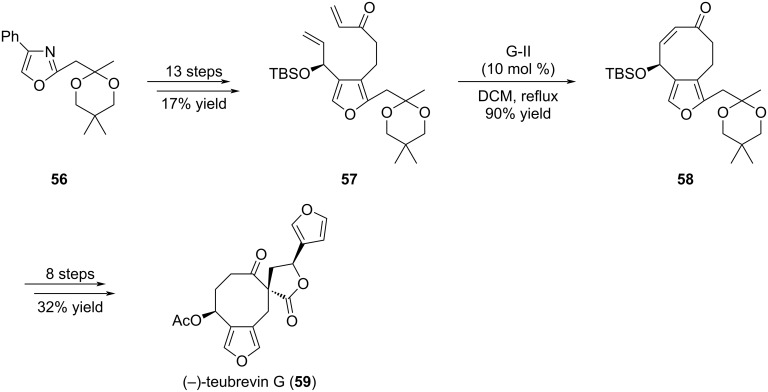
Total synthesis of (−)-teubrevin G (**59**).

**1.1.2 Other chemical series possessing a [5-8] unit; 1.1.2.1 Early-stage introduction of the eight-membered ring:** (−)-Teubrevin G (**59**) is an example of a C5-C8 bicycle in which the C5 unit corresponds to a furan ring (Scheme 9) [34]. Indeed, this compound isolated from aerial parts of *Teucrium brevifolium* has a unique structure composed of a cyclooctanone, fused with a furan ring, and bearing a spirolactone. As illustrated with the previous examples, access to the final cyclooctane ring was envisioned through a RCM reaction from an appropriate precursor. In a first attempt, compound **57**, prepared from oxazole **56**, was heated in the presence of G-I catalyst but yielded bicycle **58** only in modest amounts even after extensive reaction times (more than 3 days) and high catalyst loadings (30–35 mol %). After reaction optimization, use of G-II catalyst allowed formation of cyclooctene **58** in excellent 90% yield. This compound could ultimately be advanced to **59** in 8 steps.

**Scheme 10:**
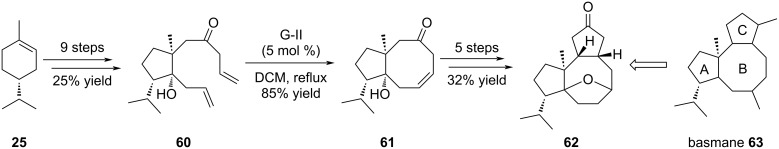
Synthesis of the core skeleton **63** of the basmane family.

Exhibiting a [5-8-5] tricyclic framework, the basmane family of terpenes differs from the fusicoccans by the arrangement of the cycles, especially the position of the shared C–C bond between ring B and ring C (Scheme 10). In 2010, Ravi et al. investigated the synthesis of the core skeleton of this family [35]. Dihydrolimonene **25** was converted in 9 steps into compound **60** before subsequent RCM. As the authors knew G-I catalyst isomerizes β,γ-unsaturated ketones, G-II catalyst was chosen to produce bicyclic compound **61**. Several additional steps were necessary to forge the last ring and obtain the core structure **63** of this series.

Schindilactone A (**68**) is a nortriterpenoid isolated from *Schisandraceae* plant family which displayed interesting biological activities in cancer and anti-infectious axis. However, access to this compound is limited because it possesses a very complex structure composed of a highly oxygenated backbone with 8 fused rings, a unique [7-8] bicyclic motif, an oxa-bridge over the cyclooctane ring, and the presence of 12 stereogenic centers [36].

**Scheme 11:**
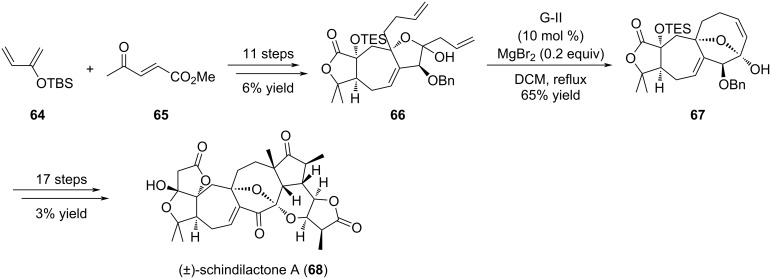
Total synthesis of (±)-schindilactone A (**68**).

The strategy proposed by the authors was to carry out a RCM reaction to elaborate the cyclooctene ring from functionalized intermediate **66**, prepared as a diastereomeric mixture (α-position of the lactone, OTES) (Scheme 11) [36]. Interestingly, in this transformation the G-II-mediated ring formation was performed in the presence of MgBr_2_, which acted as an epimerization agent at the lactol position yielding compound **67** as a single diastereomer in 65% yield. Of note, this compound entails the oxabicyclo[4.2.1]nonane core present in schindilactone A (**68**). The synthesis of schindilactone A (**68**) was achieved in 29 steps (0.11% overall yield).

**Scheme 12:**
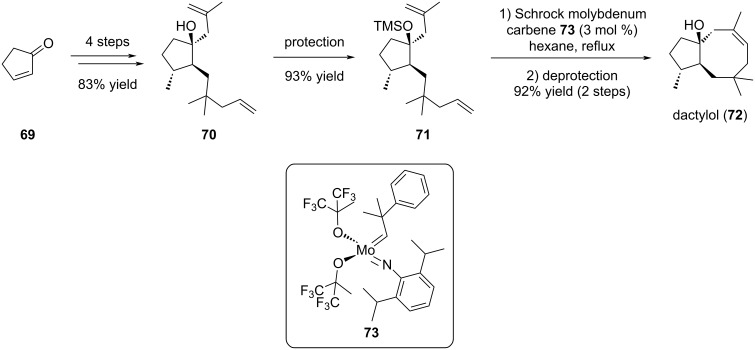
Total synthesis of dactylol (**72**).

**1.1.2.2 Late-stage introduction of cyclooctene:** The marine sesquiterpene dactylol (**72**), isolated from both sea hare *Aplysia dactylomela* and red seaweed *Laurencia poitei*, bears a rare rearranged *trans*-bicyclo[6.3.0]undecane isoprenoid skeleton. Its structure is – only – composed of a bicyclic ring with fused five- and eight-membered rings, with a hydroxy group at the junction cycle [37–38]. In 1996, Fürstner and Langemann reported an efficient and short total synthesis of the natural product dactylol (**72**) (Scheme 12) [37]. The main originality of this work was the use of the Schrock molybdenum carbene catalyst **73** for the ring-closing metathesis reaction. The metathesis precursor **70** was obtained in 4 steps from commercial cyclopentenone **69**. Protection of the alcohol function was mandatory prior to the cyclization as the free alcohol substrate failed to cyclize. As proposed by the authors, interaction between the free alcohol **70** and the molybdenum metal center may explain the reaction inhibition. Thus, after protection of the alcohol function as a silyl ether leading to diene **71**, the RCM was performed in refluxing hexane and dactylol (**72**) was isolated in 17% overall yield after silyl ether removal [18,37].

**Scheme 13:**
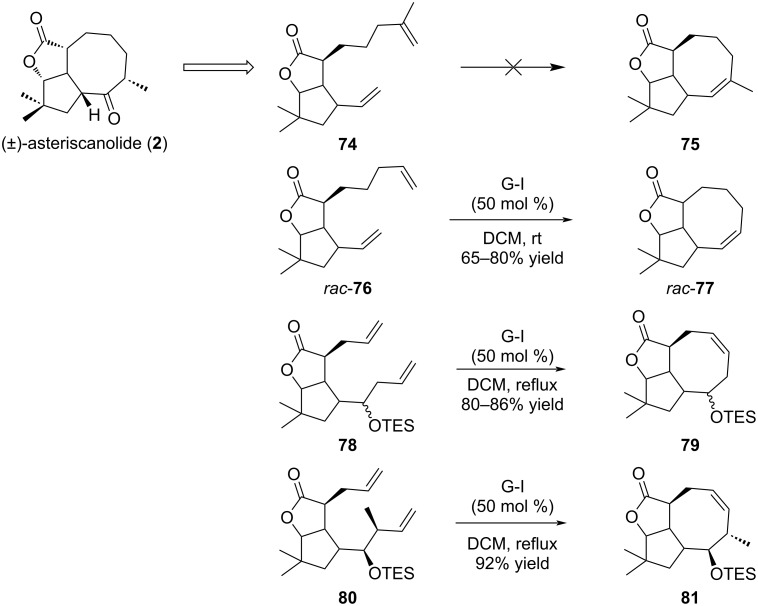
Ring-closing metathesis for the total synthesis of (±)-asteriscanolide (**2**).

Asteriscanolide (**2**), isolated in 1985 from the hexane extract of the plant *Astericus aquaticus*, is a sesquiterpene with a unique framework. Indeed, this natural compound has a rare [6.3.0] carbocyclic backbone with a bridging butyrolactone, and possesses five *cis* stereocenters [39–40]. This compound, in a racemic version, has been studied by Krafft, Cheung and Abboud (Scheme 13) [39]. The initial strategy relied on an intramolecular RCM reaction on compound **74** between the two terminal olefins, that could lead to the formation of a trisubstituted double bond (compound **75**), and further access to the required α-methyl ketone. However, despite many attempts, no cyclization occurred. Indeed, the disubstituted methylene in compound **74** and the formation of a trisubstituted alkene in **75** were assumed to cause an important steric hindrance unfavorable for the cyclization process. As a solution, the RCM was envisaged on **76** which possesses two terminal double bonds and successfully produced cyclooctene **77** in 65–80% yield with G-I catalyst in dichloromethane at room temperature. Diene **78** was also designed, with the two alkene side chains closer in length, and the cyclization produced tricyclic **79** in higher yields (80–86%). Finally, diene **80** bearing a functionalized lateral chain was submitted to RCM conditions and delivered cyclooctene **81**, an advanced intermediate in the synthesis of (±)-asteriscanolide (**2**).

**Scheme 14:**
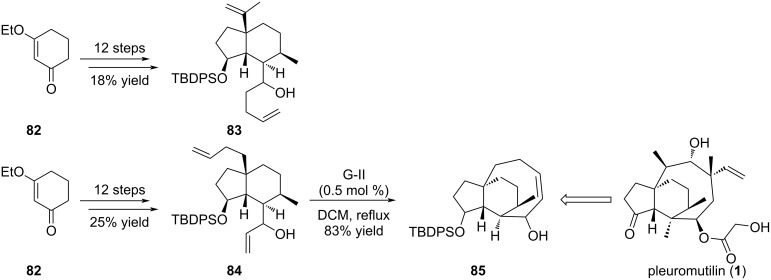
Synthesis of the simplified skeleton of pleuromutilin (**1**).

Pleuromutilin (**1**) is the flagship representative of a recent class of antibiotics which displays a propellane-like structure (Scheme 14) [41–44]. Indeed, these compounds possess a compact tricyclic backbone resulting from the fusion of a five-, six- and eight-membered ring in which the three rings share a common carbon–carbon bond. Its core structure was investigated in 2011 through the ring-closing metathesis of intermediate **83** [45]. However, the isopropenyl group seemed too hindered and the cyclization could not proceed in any investigated conditions. In a second strategy, the design of diene **84** allowed the RCM to happen and furnished tricyclic compound **85** in good yields, an advanced intermediate to the synthesis of pleuromutilin (**1**).

**Scheme 15:**
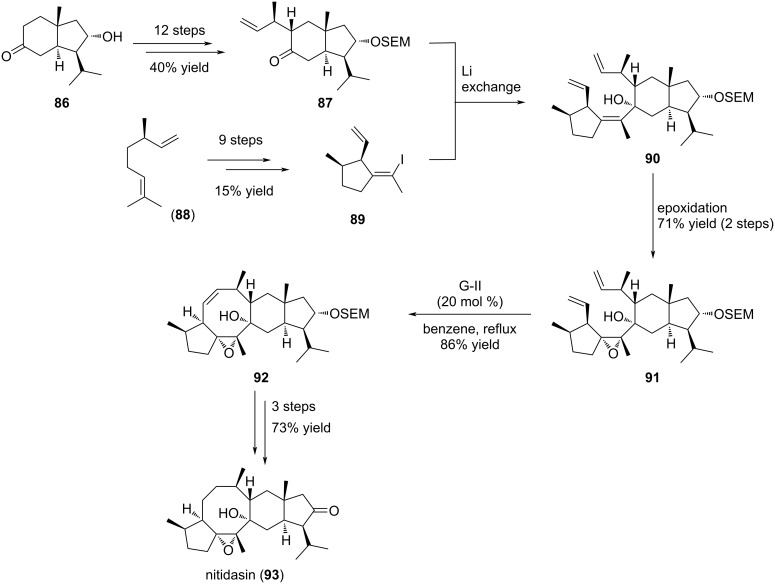
Total synthesis of (−)-nitidasin (**93**) using a ring-closing metathesis to construct the eight-membered ring.

Nitidasin (**93**) is a pentacyclic sesterterpenoid bearing a [5-8-6-5] tetracyclic all-carbon system with 10 stereogenic centers (Scheme 15). This natural product was isolated from two *Gentianella* plant species, namely *G. nitida* and *G. alborosea* used in Peruvian traditional medicine. The first report of its synthesis was given in 2014 by Hog and co-workers [46–47]. In this work, the RCM was envisaged at a late stage on advanced intermediate **90** to forge the central eight-membered ring. The synthesis begins with known hydrindanone **86** (Scheme 15). This compound was converted in 12 steps into derivative **87**, allowing introduction of a methylallyl lateral chain in α-position to the keto function. This terminal double bond prefigures the late RCM cyclization. *trans*-Hydrindanone **87** was isolated as a single diastereomer and possesses 5 out of the 10 stereogenic centers of nitidasin (**93**). In parallel, (−)-citronellene (**88**) was converted into derivative **89** possessing a terminal alkene and a vinyl iodide moiety in 9 steps. This compound can be considered as a difunctionalized compound. Indeed, the vinyl iodide function will be engaged first in a coupling reaction with hydrindanone **87** and the terminal alkene will be involved in the key RCM reaction to ensure formation of the central eight-membered ring. To achieve this goal, a model study was first conducted to define the best reaction conditions. Thus, coupling of the lithio-derivative of **89** to hydrindanone **87** proceeded smoothly to furnish the expected tertiary alcohol **90** with a high stereo- and diastereoselectivity. In a first attempt, the RCM reaction was envisioned prior to the epoxide formation. Unfortunately, no reaction occurred probably due to the presence of the tetrasubstituted olefin. Therefore, this olefin was converted into epoxide **91** and further RCM in the presence of G-II catalyst delivered the expected tetracyclic cyclooctane-bearing product **92** in high yield (86%). Of note, this cyclization occurred on a highly functionalized substrate. All the carbons of the cyclooctene ring were stereogenic, except the two of the double bond, and the precursor possesses both a hydrindane and a cyclopentane unit. The final steps of this synthesis involved alcohol deprotection, double bond hydrogenation and oxidation, and allowed the total synthesis of (−)-nitidasin (**93**) in 27 steps.

**Scheme 16:**
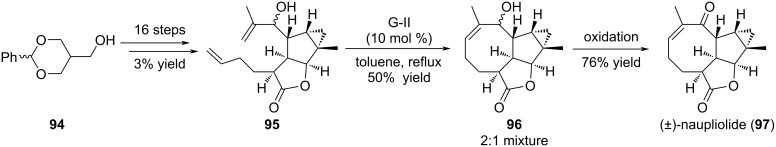
Total synthesis of (±)-naupliolide (**97**).

Naupliolide (**97**) was first isolated from the aerial parts of *Nauplius graveolens* in 2006. This tetracyclic natural product displays a similar backbone as asteriscanolide (**2**), with an α,β-unsaturated ketone on the eight-membered ring, fused with a γ-butyrolactone and a cyclopentane, onto which a cyclopropane is also grafted [48]. The total synthesis of (±)-naupliolide (**97**) reported by Ito et al. constituted another example of the usefulness of the RCM to construct a cyclooctane ring fused to a cyclopentane unit and presenting a *trans-*ring junction (Scheme 16) [48]. The synthesis started from compound **94** which was converted in 16 steps into derivative **95**, the precursor of the RCM. The cyclization was performed in refluxing toluene in the presence of G-II catalyst and provided tetracyclic compound **96** with 50% yield. In this case, the RCM involved a terminal diene and a disubstituted diene. Further oxidation of the secondary alcohol furnished naupliolide (**97**).

#### 1.2 Enyne ring-closing metathesis

The enyne ring-closing metathesis (EYRCM) reported by Katz in 1985, represents an attractive variant of the classical RCM with the replacement of one of the alkenes by an alkyne function. Thus, EYRCM is atom economic and provides a 1,3 diene, which constitutes an ideal partner for further functionalization, typically a Diels–Alder process [4,8,10].

**Scheme 17:**
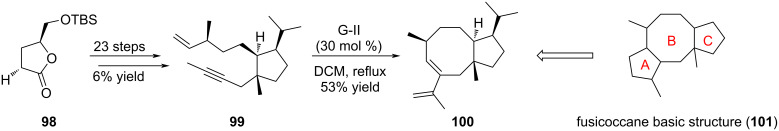
Synthesis of the A-B ring structure of fusicoccane (**101**).

In 2008, Patrick et al. investigated the access to bicyclic structure **100** mimicking the A-B ring system of the fusicoccane series (Scheme 17) [49]. Starting from lactone **98**, enyne **99** was prepared in 23 steps. The EYRCM was planned at the final stage allowing an access to compound **100**. Despite the relative simplicity of substrate **99**, the reaction was running in the exclusive presence of G-II catalyst and necessitated a high catalyst loading (30 mol %) to eventually furnish modest yields. The presence of two major byproducts was also noticed, namely the cross metathesis adduct resulting from 2 equivalents of **99** and the cross metathesis adduct resulting from **99** and a styrene unit coming from the catalyst. In addition, the corresponding RCM on the alkene analogue of **99** did not proceed either with G-II or Schrock catalysts, showcasing the substrate sensitivity of this reaction.

#### 1.3 Tandem ring-closing metathesis

The tandem ring-closing metathesis (TRCM) approach offers the opportunity to form two contiguous cycles in one step from a well-designed precursor. To this end, the starting substrate should integrate a dienyne moiety in its backbone. One of the olefins reacts first with the alkyne to form a carbocycle possessing a vinyl moiety, which in turn reacts with the second alkene, thus producing the expected bicyclic structure in a tandem process.

**Scheme 18:**
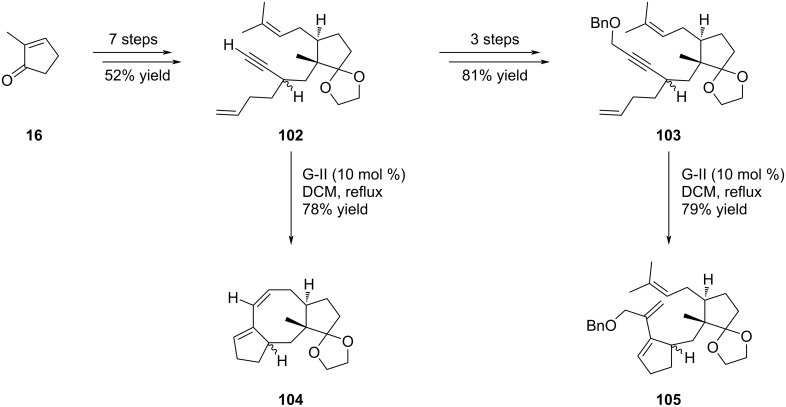
First attempts of TRCM of dienyne substrates.

Ophiobolin A (**8**), one of the representatives of the ophiobolin family, contains a [5-8-5] tricyclic backbone with a side chain on the D-ring. It was isolated in 1958 from the fungus *Ophiobolus miyabeanus*, and displayed a remarkable cytotoxicity against several cancer cell lines [50]. Meanwhile, variecolin (**3**) was isolated from the fermentation broth of the fungus *Aspergillus variecolor* in 1991, and it appears to be a potent immunosuppressant. Structurally, variecolin (**3**) has a complex tetracyclic backbone with a fused [5-8-6-5] skeleton, with 8 stereocenters, 4 of them being contiguous and located on the C-ring [50]. The synthesis of the core structures of ophiobolin A (**8**) and variecolin (**3**) was undertaken by Gao and his group who used this strategy to construct both A and B rings (Scheme 18) [50]. 2-Methylcyclopentenone **16** was selected as a suitable A-ring starting material for the synthesis of ophiobolin A (**8**) (Scheme 18). Sequential modulations resulted in dienyne intermediates **102** and **103**, the latter presenting a functionalized alkyne moiety prefiguring the aldehyde function of ophiobolin A (**8**). These two compounds were submitted to the EYRCM in the presence of G-II catalyst and furnished two different outcomes. Indeed, compound **102** gave the expected product **104** in 78% yield, whereas precursor **103**, bearing hindered *gem*-dimethyl and benzyl protecting group, did not undergo the tandem process and furnished the monocyclized product **105**.

**Scheme 19:**
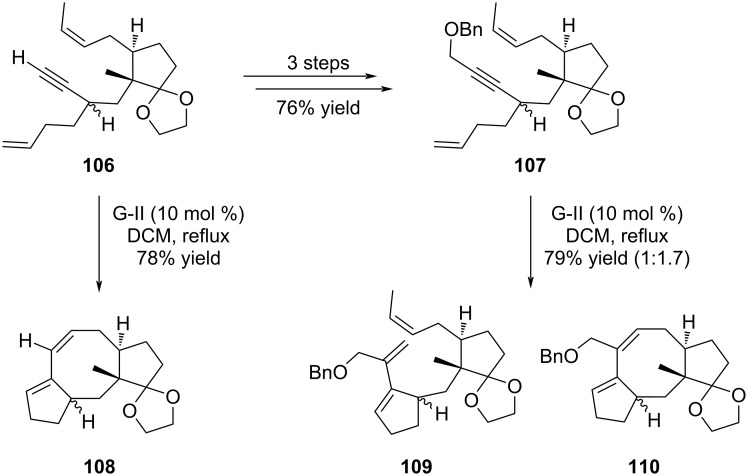
TRCM on optimized substrates towards the synthesis of ophiobolin A (**8**).

To circumvent this monocyclization reaction, Gao and co-workers decided to simplify the *gem*-dimethyl group by a methyl residue and prepared the analogous compounds of **102** and **103**, namely **106** and **107**, respectively (Scheme 19). With these compounds in hand, terminal alkyne **106** provided the desired product **108** as previously observed with **102**, while the protected alkyne **107** furnished a 1:1.7 mixture of monocyclized **109** and the expected product **110** in overall good yield. This study highlighted that a less-hindered olefin facilitated the second enyne metathesis of this tandem reaction.

**Scheme 20:**
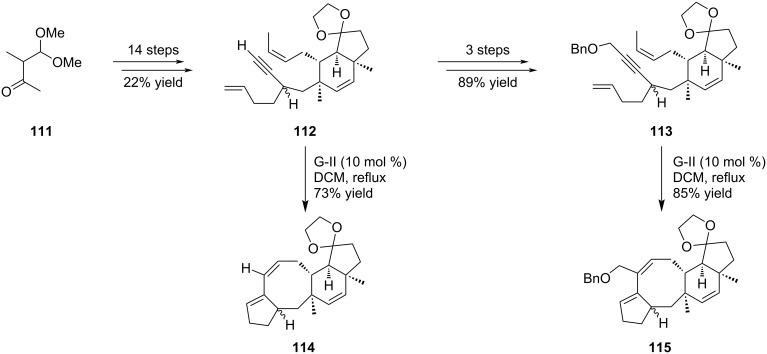
Tandem ring-closing metathesis for the synthesis of variecolin intermediates **114** and **115**.

The same strategy was applied for the synthesis of the variecolin [5-8-6-5] skeleton (Scheme 20). Dienynes **112** and **113** were first prepared from methyl ketone **111** and subsecuently submitted to the metathesis conditions in the presence of G-II catalyst. Interestingly, in both cases the expected compounds resulting from the TRCM were obtained in good yields. Of particular interest, precursor **113** furnished **115** as the sole product.

#### 1.4 Allylsilane ring-closing metathesis

**Scheme 21:**
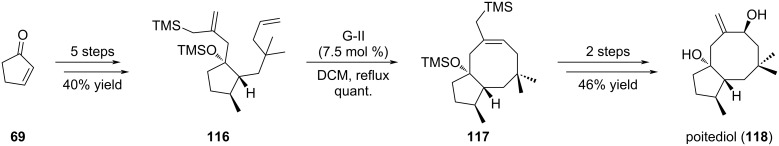
Synthesis of poitediol (**118**) using the allylsilane ring-closing metathesis.

As demonstrated above and in the literature [4–10], the RCM reaction constitutes a powerful tool to construct rings of various sizes. In 2009, Vanderwal and Dowling extended this process to the use of allylsilane derivatives which can be further submitted to an electrophilic desilylation reaction [51–54] giving an access to *exo*-methylene [38,55]. This concept was exemplified with the synthesis of poitediol (**118**) (Scheme 21), a bicyclopentacyclooctane compound isolated from the red seaweed *Laurencia poitei* in 1978. This sesquiterpene bearing a [5-8] bicyclic structure includes an *exo*-methylidene moiety on the eight-membered ring [38,55]. Thus, allylsilane diene **116** prepared from cyclopentenone **69** as a 4:1 diastereomeric mixture was submitted to RCM conditions in the presence of G-II catalyst in refluxing dichloromethane. The bicyclic product **117** was obtained in quantitative yield. Given the amount of electrophilic desilylation agents which can be used, a large diversity of compounds can be accessible through this method [51–52]. Oxidation followed by desilylation with fluoride source produces the natural product poitediol (**118**) in overall good yield.

### 2 Nozaki–Hiyama–Kishi (NHK) cyclization

The Nozaki–Hiyama–Kishi (NHK) reaction is an interesting coupling reaction involving the addition of a halogeno derivative (either bromide or iodide) to an aldehyde in the presence of nickel and chromium salts, typically NiCl_2_/CrCl_2_, generating an alcohol. In its original version, the stereochemistry of the adduct was not controlled, yielding a mixture of stereoisomers. Recent developments were undertaken to propose an asymmetric version [56–57].

In its intramolecular version, the NHK reaction was successfully applied by Kishi in 1989 to forge the cyclooctane ring of ophiobolin C (**30**) [5-8-5] framework, thus opening the door for further applications [58]. Indeed, several groups included this method at a late stage in their synthetic plan.

#### 2.1 Synthesis of pleuromutilin

**Scheme 22:**
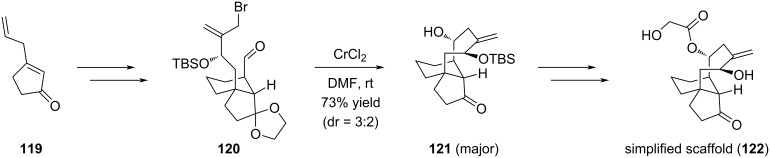
Access to scaffold **122** by a NHK coupling reaction.

The purpose of Sorensen's work was to design an efficient strategy allowing a rapid access to the pharmacophoric core of pleuromutilin, e.g., compound **122** for further derivatization and structure–activity relationship (SAR) studies [59]. In this case, an intramolecular NHK was carried out on compound **120** prepared from 3-allylcyclopent-2-enone (**119**) to give the tricyclic compound **121** possessing the eight-membered ring in 73% yield as the major compound. The diastereoselectivity was modest with a dr of 3:2. Esterification of the secondary alcohol followed by silyl removal gave access to the key scaffold **122** (Scheme 22).

#### 2.2 Synthesis of aquatolide (**4**): Late-stage NHK medium-ring formation

**Scheme 23:**
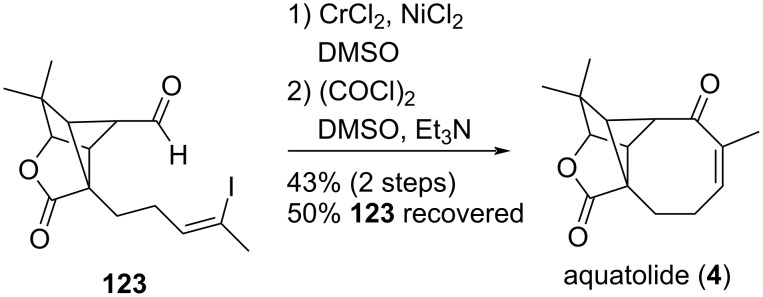
Key step to construct the [5-8] bicyclooctanone core of aquatolide (**4**).

Aquatolide (**4**) is a sesquiterpene lactone isolated from *Astericus aquaticus* (Asteraceae) which possesses a unique backbone featuring a cyclobutane moiety fused to a cyclopentane ring, a γ-lactone, and an eight-membered enone [60–61]. Zhang and Gu achieved its preparation through the use of a late-stage intramolecular NHK reaction (Scheme 23) [62]. Thus, when compound **123** was treated with CrCl_2_ in the presence of a catalytic amount of NiCl_2_ in DMSO and further oxidized under Swern conditions, aquatolide (**4**) was obtained in 43% yield over the two steps along with 50% recovery of the starting material **123**.

**Scheme 24:**
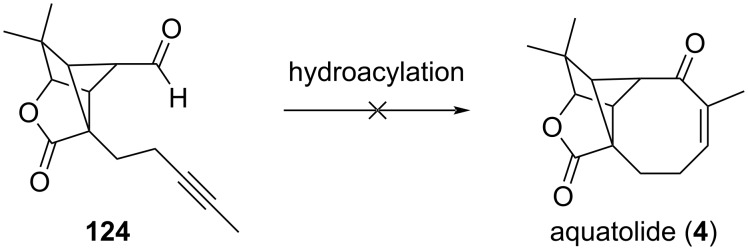
Initial strategy to access aquatolide (**4**).

Of note, the initial strategy involved an intramolecular hydroacylation on alkynyl compound **124** to construct the cyclooctanone unit. Despite several attempts, no cyclization occurred (Scheme 24).

### 3 Pd-mediated cyclization

#### 3.1 Pd-promoted intramolecular alkenylation of methyl ketone: synthesis of cotylenin A (**130**)

**Scheme 25:**
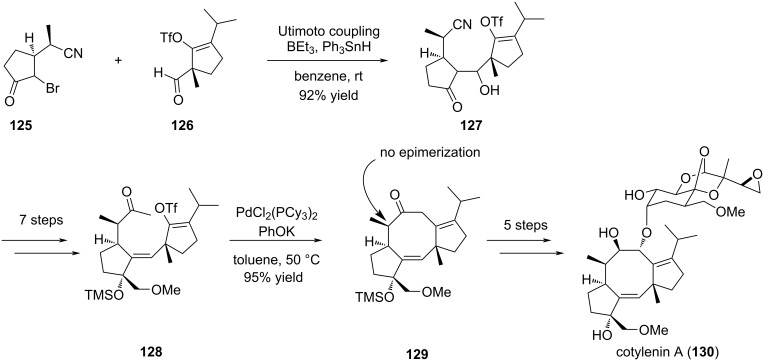
Synthetic plan to cotylenin A (**130**).

Nakada exploited the usefulness of a Pd-promoted intramolecular alkenylation of methyl ketone to forge in the late-stage of the synthesis the eight-membered ring of cotylenin A (**130**) [63]. To this end, the two cyclopentane units of the tricyclic core of cotylenin A were prepared separately and first assembled by an Utimoto coupling reaction to give after additional functionalization steps the key intermediate **128**. This compound constituted the substrate for the Pd-promoted intramolecular cyclization. In this case, an enol triflate was used instead of an alkenyl halide which required the presence of an electron-rich phosphine, a lower temperature (50 °C instead of 100 °C) to avoid C-7 epimerization, and two equivalents of the Pd complex. The cycloadduct **129** was obtained in very high yield and could be converted to cotylenin A (**130**) in 5 steps. This work constituted an enantioselective total synthesis of cotylenin A (**130**) (Scheme 25).

#### 3.2 Intramolecular Mizoroki–Heck reaction: construction of the [5-8] bicyclic ring system of brachialactone

**Scheme 26:**
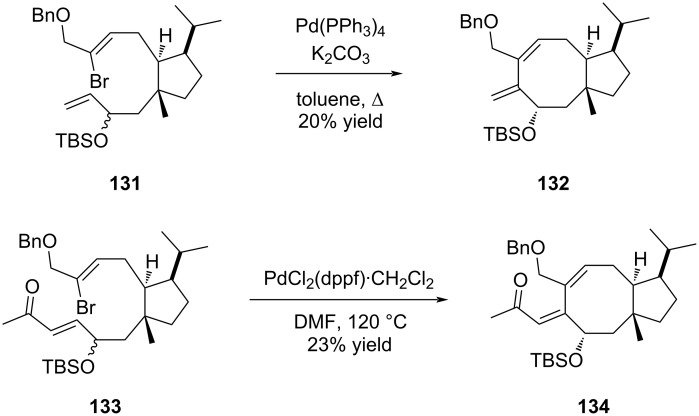
[5-8] Bicyclic structure of brachialactone (**7**) constructed by a Mizoroki–Heck reaction.

**Scheme 27:**
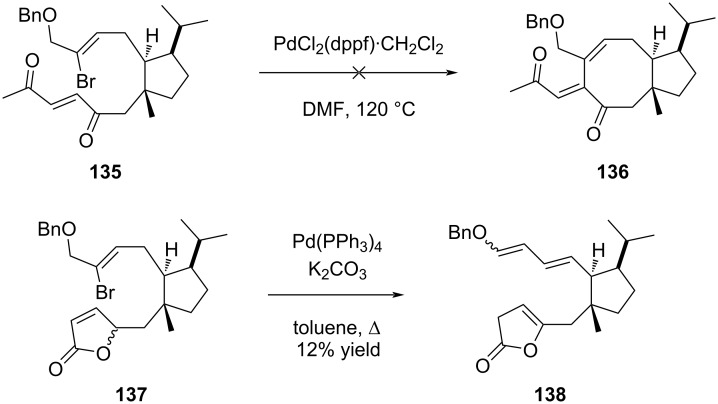
Influence of the replacement of the allylic alcohol moiety.

A study of the versatility of the Mizoroki–Heck reaction in an intramolecular version was recently described by Nishikawa to elaborate the eight-membered ring of brachialactone (**7**) (fusicoccane series) from an advanced cyclopentane intermediate accessed from (*S*)-limonene [64]. Several cyclopentane precursors were prepared to investigate the importance of the alkene functionalization on the cyclization. Thus, the reaction proceeded in the presence of silylated allylic alcohol **131**, producing **132** albeit in low yield. The presence of a terminal methyl ketone function did not influence the reaction outcome as compound **133** produced cycloadduct **134** in the same low-moderate yield. Interestingly, the reaction was highly selective and only one single diastereomer was formed (Scheme 26). However, replacement of the allylic alcohol by a vinyl ketone (compound **135**) or a butenolide (compound **137**) moiety dramatically influenced the outcome of the reaction and no cycloadduct was observed in both cases (Scheme 27).

### 4 Radical cyclization (including SmI_2_)

Introduced by Kagan more than four decades ago, samarium diiodide (SmI_2_) has found multiple applications in natural product synthesis [65]. Employed in C–C bond-formation reactions, this single-electron reducing agent has been particularly useful for five- to eight-membered ring cyclizations [65]. Its tunable reactivity opens access to both radical and anionic processes, hence favoring reactivity with various types of substrates, ranging from halides to carbonyls and alkenes/alkynes. It is comprehensible that this reagent attracted early interest in natural product synthesis and more precisely on medium-sized ring formation.

#### 4.1 SmI_2_-mediated Barbier-type ring annulation towards variecolin synthesis

**Scheme 28:**
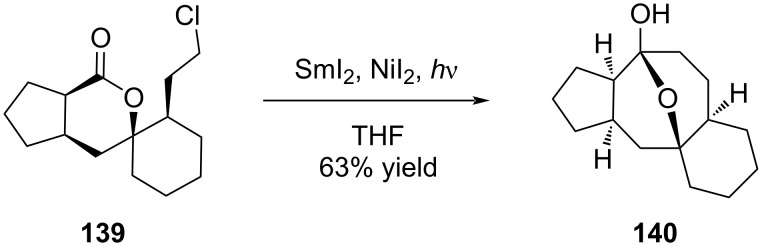
Formation of variecolin intermediate **140** through a SmI_2_-mediated Barbier-type reaction.

Following Kagan’s work, Molander’s group thoroughly investigated the potential of SmI_2_-mediated intramolecular ring closure. Among the approaches they studied, the Barbier-type cyclization rapidly gained popularity for five-to-eight-membered ring formation. Based on a reductive addition of an alkyl halide to a carbonyl group, implementation of the Barbier-type ring closure relied thus on the preliminary introduction of both aldehyde and alkyl halide functional groups on a suitable substrate. The mechanism was first thought to involve the coupling of an alkyl radical and a ketyl radical, but is now assumed to proceed through the formation of an organosamarium intermediate, resulting from two successive single-electron reductions [66]. This strategy was successfully applied to the construction of variecolin intermediate **140** possessing a [5-8] ring system in its backbone (Scheme 28). Interestingly, the reaction was carried out under UV irradiation and in the presence of catalytic nickel diiodide allowing formation of the expected compound in 63% yield [67].

#### 4.2 SmI_2_-mediated ketyl addition: discussion around pleuromutilin scaffold access

**Scheme 29:**
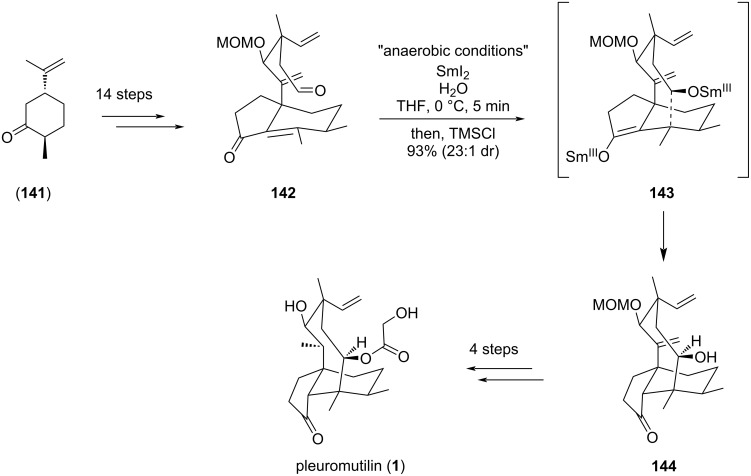
SmI_2_-mediated ketyl addition. Pleuromutilin (**1**) eight-membered ring closure via C^5^–C^14^ bond formation.

Despite the promising results of the SmI_2_-mediated ketyl addition, one would have to wait almost two decades before employing it again in eight-membered ring closure. Among the diterpenes and sesterterpenes featuring a cyclooctane framework, (+)-pleuromutilin (**1**) was recently targeted by Reisman’s group through an elegant approach involving a SmI_2_-mediated cyclization as a key step of the synthesis [68]. Indeed, this pivotal annulation step was conducted on aldehyde **142** prepared from (+)-*trans*-dihydrocarvone (**141**) and yielded the expected complex tricyclic [5-8-6] alcohol **144** in 93% with a high diastereoselectivity (dr 23:1). Of note, the reaction conditions were subjected to extensive studies for optimization. The intermediate hydrindanone enal fragment **142**, bearing both an aldehyde and an alkene function, was subjected to rigorously anaerobic conditions, in the presence of 3 equivalents of SmI_2_, 6 equivalents of H_2_O, and TMSCl as a quenching agent (Scheme 29).

**Scheme 30:**
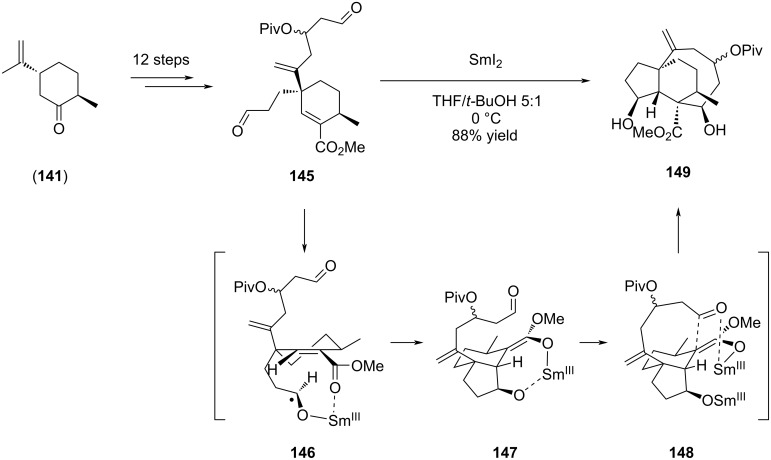
SmI_2_-mediated dialdehyde cyclization cascade of [5-8-6] pleuromutilin scaffold **149**.

In their discussion, the authors highlighted the differences with previously reported SmI_2_-mediated eight-membered ring closure in pleuromutilin (**1**) synthesis. Indeed, in 2013, Procter’s group was actually the first to report a 34-step enantiospecific total synthesis of (+)-pleuromutilin (**1**), where the eight-membered ring was accessed through a SmI_2_-mediated cyclization cascade reaction of a dialdehyde [69]. In this approach, an original way was proposed to form in a single step the tricyclic core of pleuromutilin (**1**) with a stereocontrol at the four contiguous stereocenters [69–70]. The dialdehyde was obtained from *trans*-dihydrocarvone (**141**) and treated early in the sequence (step 13/34) by SmI_2_. The authors assumed that the cascade reaction was initiated with the left-hand aldehyde ketyl formation **146** which further attacked the alkene and oriented the *anti*-5-*exo*-*trig*-cyclization toward (*Z*)-Sm^III^ enolate **147**. The cyclooctane ring was then accessed through the (*Z*)-Sm^III^ enolate aldol cyclization. The different organosamarium species generated during the cascade cyclization mechanism were hypothesized to drive the diastereoselectivity on each of the stereocenters created during the process (Scheme 30).

#### 4.3 Nickel-catalyzed reductive cyclization: mutilin and pleuromutilin C12-epimers access

**Scheme 31:**
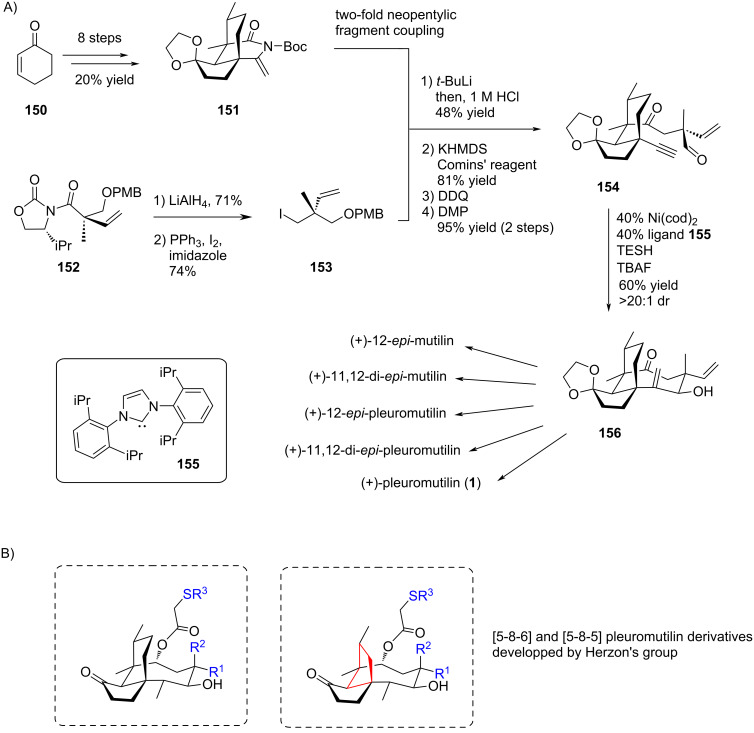
A) Modular synthetic route to mutilin and pleuromutilin family members by Herzon’s group. B) Scaffolds of pleuromutilin derivatives reported by Herzon’s group.

In view of maximizing the scope of accessible pleuromutilin epimers from one single synthetic pathway, Herzon’s group reported a modular and convergent route, where the eight-membered ring was built intramolecularly in a late-stage step (Scheme 31A) [71–72]. With this strategy, the authors intended to benefit from the pre-installed quarternary carbon centers or sp^2^-hybridized carbons to limit the number of rotatable bonds and transannular *syn*-pentane-type interactions, hence to lower the entropic penalty associated to ring closure. To reach this goal, they first dedicated efforts in optimal syntheses of an electrophilic enimide **151** [73] and its conjunctive reagent, the neopentyl iodide **153**. Both fragments were then engaged in a two-fold neopentylic fragment coupling, giving a bicyclic intermediate which after appropriate modification led to the alkynyl aldehyde cyclization precursor **154**. The ring closure was performed under catalytic reductive conditions in the presence of Ni(cod)_2_ and ligand **155** as the catalytic system, and triethylsilane as a reductant. The expected cycloadduct was obtained as a single diastereomer (dr > 20:1) in 60% yield [71]. This intermediate allowed then access to several members of the mutilin/pleuromutilin family: total syntheses of (+)-12-*epi*-mutilin, (+)-11,12-di-*epi*-mutilin, (+)-12-*epi*-pleuromutilin, (+)-11,12-di-*epi*-pleuromutilin, and (+)-pleuromutilin (**1**) itself were there described in 17 to 20 steps [71–72]. The series was later extended via a revised and improved synthetic strategy toward the tricyclic [5-8-6]-pleuromutilin platform [74], allowing access to remarkable chemical diversity for pleuromutilin derivatives which were evaluated against a panel of Gram-positive and Gram-negative bacteria (Scheme 31B).

#### 4.4 Photocatalyzed oxidative ring expansion: alternative radical chemistry for pleuromutilin scaffold construction

**Scheme 32:**
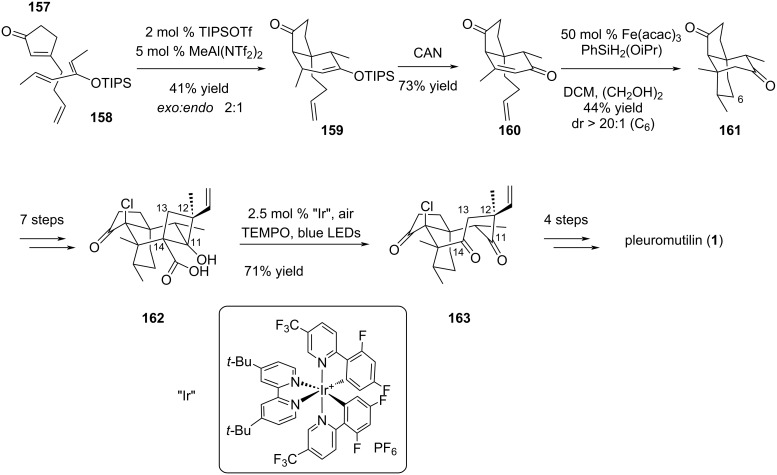
Photocatalyzed oxidative ring expansion in pleuromutilin (**1**) total synthesis.

Following the advent of photoredox catalysis in ring-opening and ring-expansion chemistry [75], a new route was proposed by Foy and Pronin to access the cyclooctane unit of the pleuromutilin scaffold [76]. In this very recent paper, the authors implemented adequately chosen tactics allowing the completion of the 15-step and 16-step synthesis of mutilin and pleuromutilin (**1**), respectively. To access the terpenoid core, the synthetic pathway was articulated around a ring expansion of a fused cyclobutane/perhydroindanone fragment, leading to the cyclooctane motif. Interestingly, and unlike the previously reported strategies, the perhydroindanone precursor was prepared starting from achiral building blocks **157** and **158**. They were engaged in a selective *exo* Diels–Alder cycloaddition, which resulted in compound **159**. The enol ether was oxidized by ceric ammonium nitrate (CAN) to deliver intermediate **160**, which was further subjected to an iron-catalyzed hydrogen atom transfer generating tricyclic intermediate **161**. Further functionalization permitted the formation of the fused cyclobutane acid **162** as the desired precursor for the cyclooctane formation. The ring expansion was achieved in the presence of an iridium catalyst and under blue LED irradiation, via the trapping by TEMPO or O_2_ of the cyclobutyl radical resulting from decarboxylation, which allowed a Criegee- or a Grob-type fragmentation to generate cyclooctadione **163**. Pleuromutilin (**1**) was then accessed in 4 additional steps (Scheme 32).

#### 4.5 Reductive radical cascade cyclization: toward total synthesis of (−)-6-*epi*-ophiobolin N (**168**) and (+)-6-*epi*-ophiobolin A (**173**)

**Scheme 33:**
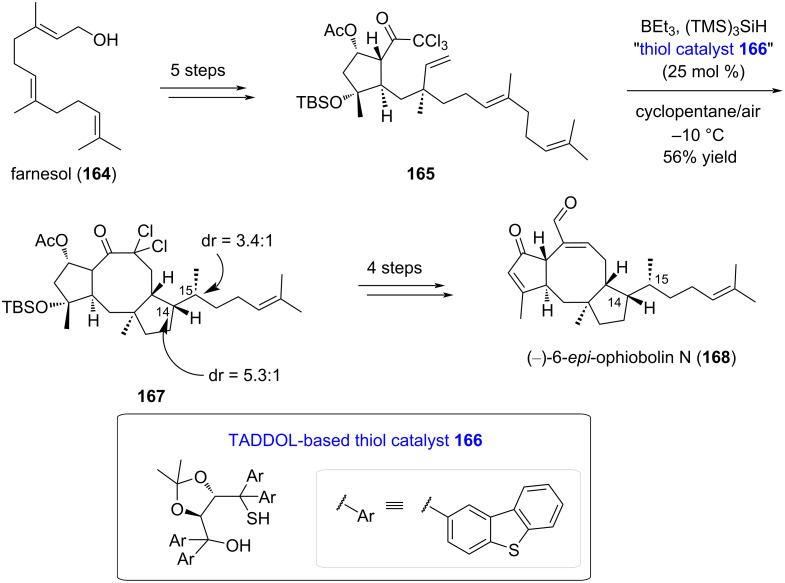
Reductive radical cascade cyclization route towards (−)-6-*epi*-ophiobolin N (**168**).

Despite indisputable success of the radical approach in the formation of the eight-membered ring of pleuromutilin (**1**), the chemistry was scarcely extended to other terpenoid molecules. Only ophiobolin synthetic strategy was implemented with an 8-*endo*/5-*exo* radical cascade cyclization by Maimone’s group [77]. Inspired by the cyclase-mediated polyterpene cyclization mechanism, they proposed a rapid 9-step strategy starting from abundant monoterpene farnesol (**164**), chemically converted into cyclopentanol **165** bearing a trichloroketone function that is a well-known radical precursor. The authors found that the correct stereochemistry of the stereogenic centers formed during the cascade cyclization was secured by the use of benzothiophene-based TADDOL thiol **166** as chiral catalyst. They obtained in one single step a 5.3:1 and 3.4:1 diastereomeric ratio for C14 and C15, respectively, while forming the desired *trans* [5-8] ring junction in C10-C11 (Scheme 33). (−)-6-*epi*-Ophiobolin N (**168**) was then accessible with four additional steps.

**Scheme 34:**
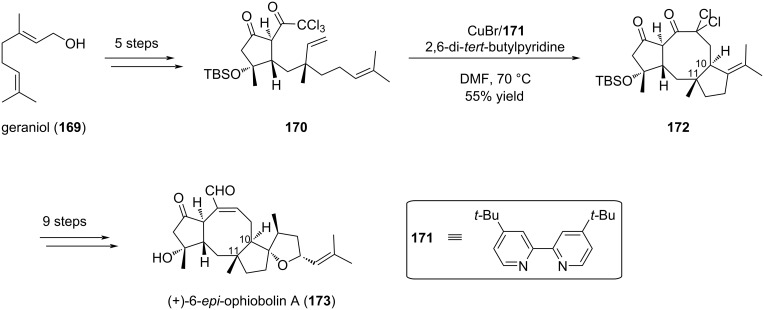
Reductive radical cascade cyclization route towards (+)-6-*epi*-ophiobolin A (**173**).

Following the extensive work they dedicated to (−)-6-*epi*-ophiobolin N (**168**), Maimone’s group reused one of the investigated cyclization conditions for the total synthesis of (+)-6-*epi*-ophiobolin A (**173**), which was then described in a 14-step strategy, starting from geraniol (**169**) [78]. Unlike compound **168**, the installation of stereogenic centers at C14 and C15 was not sought at the 8-5 cyclization stage of the strategy, since formation of the tetrasubstituted alkene provided an adequate precursor towards the tetrahydrofuran-ring formation. The intramolecular atom-transfer radical cyclization was thus carried out with achiral di-*tert-*butylbipyridine-ligated Cu^I^ **171** to generate the [5-8-5] scaffold **172** in 55% yield, while securing the C10 stereocenter in the required configuration and providing a suitable alkene bond for further functionalization. (+)-6-*epi*-Ophiobolin A (**173**) was eventually accessed with 9 additional steps (Scheme 34).

#### 4.6 Other radical approaches towards eight-membered ring closure

**Scheme 35:**
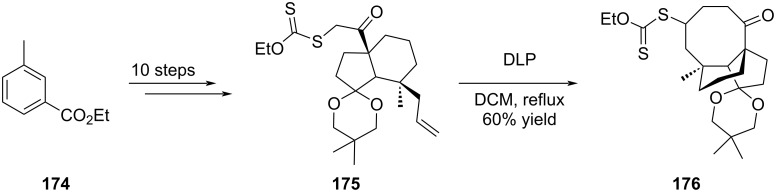
Radical 8-*endo-trig*-cyclization of a xanthate precursor.

As seen above, radical-based pathways proved to be efficiently implemented in peculiar pleuromutilin tricyclic scaffold construction. Alternative reagents were thus explored to close the eight-membered ring via initial radical genesis. For instance, Bacqué et al. reported the insertion in the cyclization precursors of functional groups prone to form radicals (Scheme 35). In their investigations toward pleuromutilin scaffold synthesis, they proposed to perform a radical 8-*endo*-*trig*-cyclization of a xanthate precursor. The xanthate group was quantitatively installed on an adequately functionalized hydrindanone starting moiety **175**, obtained in 10 steps from ethyl *m*-toluate (**174**), which was then refluxed in the presence of a small amount of dilauroyl peroxide (DLP) as radical initiator. The eight-membered ring **176** was obtained in 60% yield as a single diastereomer [79].

### 5 Pauson–Khand reaction

**Figure 5:**
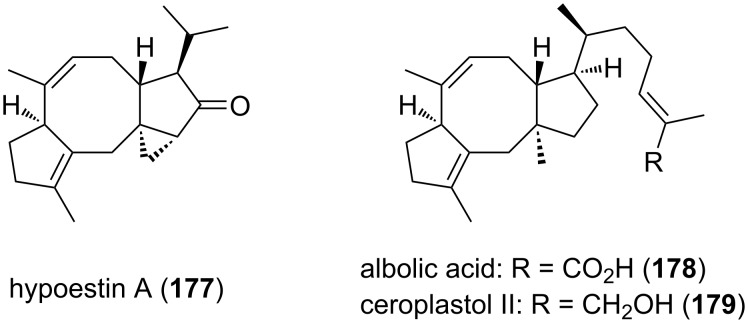
Structural representations of hypoestin A (**177**), albolic acid (**178**), and ceroplastol II (**179**) bearing the same [5-8-5] core structure.

Discovered in the seventies [80], the Pauson–Khand reaction has been widely used for the formation of cyclopentenone motifs, by reaction of an alkene, alkyne, and carbon monoxide. This is only recently, in April 2022, that this reaction was reported to allow the formation of an eight-membered ring [81]. Indeed, Li and co-workers employed the Pauson–Khand reaction in order to undertake the total synthesis of hypoestin A (**177**), albolic acid (**178**), and ceroplastol II (**179**) (Figure 5) bearing the same [5-8-5] tricyclic core structure.

Belonging to the fusicoccane diterpenoid family, hypoestin A (**177**) (Figure 5) can play an important role in cardiovascular and neurological diseases by its inhibitory activity of the Ca_v_3.1 calcium channel. From a chemical point of view, hypoestin A (**177**) presents a complex structure: it is composed of a [5-8-5-3] tetracyclic core skeleton, with five stereogenic centers, whose four of them are contiguous. Before their work, no asymmetric total synthesis of hypoestin A (**177**) has been reported. During this study, they also looked into the asymmetric total synthesis of albolic acid (**178**) and ceroplastol II (**179**). Only one paper reported their total synthesis, using a reductive coupling of aldehydes for the synthesis of the eight-membered ring with an excellent yield of 96% [82]. However, the total synthesis included 24 steps, with an overall yield of 5% and 4% for albolic acid (**178**) and ceroplastol II (**179**), respectively. To synthesize these 3 complex natural products, they used the same method to access the [5-8-5] tricyclic advanced intermediate **184**. After that, different strategies allowed the functionalization for each natural product. Their general method consisted in using an intramolecular Pauson–Khand reaction catalyzed by a rhodium complex from an allene-yne substrate to build the eight-membered ring, which was quite challenging.

**Scheme 36:**
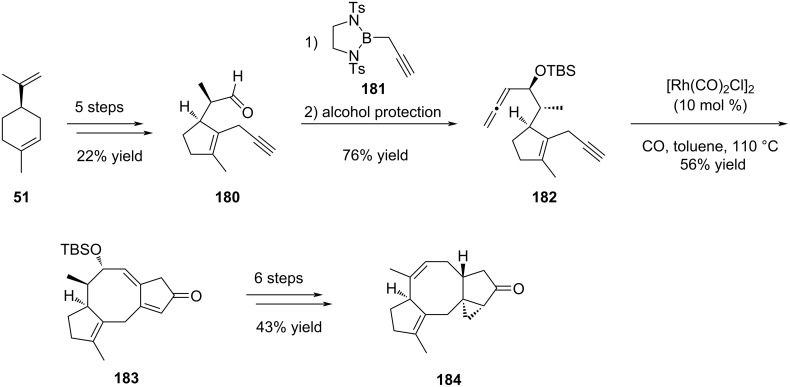
Synthesis of the common [5-8-5] tricyclic intermediate of hypoestin A (**177**), albolic acid (**178**), and ceroplastol II (**179**).

Starting from commercial (*R*)-limonene (**51**), Li and co-workers synthesized allene-yne intermediate **182** (Scheme 36) in 7 steps, with a 16% overall yield. To access the allene moiety **182**, they first tried addition of propadienyllithium to aldehyde **180**, but they obtained a complex mixture of allenic alcohol and homopropargylic alcohol. Then, they were inspired by Corey’s work [83] and formed this key function through the addition of propargylborane **181** to aldehyde **180**, with a good 80% yield. Protection of the obtained alcohol was also necessary. All these 7 first steps set the stereochemistry of the methyl group on the further eight-membered ring and the hydrogen atom at the junction of the five- and eight-membered rings.

The next step of the synthesis was the key formation of the eight-membered ring and several attempts were necessary in order to determine the optimal conditions. They first explored the nature of the catalyst among different rhodium-based catalysts and 5% of [Rh(CO)_2_Cl]_2_ were found to be the best catalyst loading (28% yield). The addition of a silver additive, to make the reaction quicker and to remove a CO ligand, appeared to be useless in this case, with no reaction, and the use of 50% dppp as ligand allowed the formation of the product but with lower yields (25%). The effect of the concentration was also important, since increasing or lowering the concentration from 0.01 M resulted in lower yields. The same trend was observed when the temperature was varied. Finally, the amount of catalyst was explored: increasing from 5 mol % to 10 mol % almost doubled the yield (respectively 38% and 60% yield), and no significant increase in the yield was found when using 20% of catalyst (62% yield). Thus, the use of 10 mol % of [Rh(CO)_2_Cl]_2_ catalyst in toluene at 110 °C and under CO atmosphere yielded the cyclized compound **183** in 56%, even at large scale (2 g). Having this tricyclic structure in hands, several more steps were needed to access advanced intermediate **184** for the synthesis of both natural products. Thus, they first selectively hydrogenated the double bond on the eight-membered ring, and ketone reduction allowed to install the cyclopropane ring thanks to the allylic alcohol. The latter was submitted to Ley oxidation, then deprotection and removal of the obtained alcohol gave the [5-8-5] advanced intermediate **184** (Scheme 36).

**Scheme 37:**
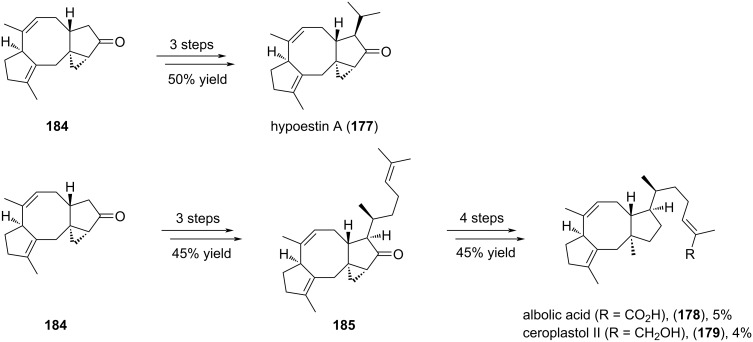
Asymmetric synthesis of hypoestin A (**177**), albolic acid (**178**), and ceroplastol II (**179**).

The stage was set for the further functionalization to synthesize hypoestin A (**177**), albolic acid (**178**), and ceroplastol II (**179**) (Scheme 37). For the synthesis of hypoestin A (**177**), the side chain was introduced by selective deprotonation of **184**, addition of acetaldehyde, dehydration and conjugated addition of Me_2_CuLi. These three last steps provided the desired product **177** in 50% overall yield. For the synthesis of albolic acid (**178**) and ceroplastol II (**179**), the side chain was introduced by deprotonation and addition of the corresponding aldehyde. After dehydration of the subsequent alcohol and conjugated addition of Me_2_CuLi, the regioselective reductive opening of cyclopropane **185** was performed with lithium in liquid ammonia in order to introduce the angular methyl group. In the same time, the ketone was reduced into an alcohol, which one was submitted to Barton deoxygenation. The alkene side chain underwent a metathesis reaction with methyl methacrylate to introduce an ester. Finally, this latter was hydrolyzed or reduced to respectively provide albolic acid (**178**) and ceroplastol II (**179**).

**Figure 6:**
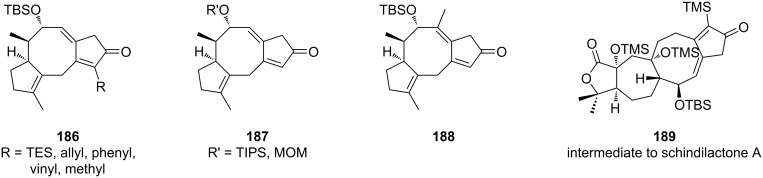
Scope of the Pauson–Khand reaction.

The scope of the reaction was also extended to various products containing the [X-8-5] tricyclic system (Figure 6). Several functionalized terminal alkynes succeeded in the Pauson–Khand cyclization (**186**), with moderate yields ranging from 40 to 61%. It was also possible to change the alcohol protecting group (**187**) without significant change in yields (45–62%). The addition of a methyl group on the eight-membered cycle (**188**) was also possible through the functionalization of the internal position of the allene (51%). In the same conditions, an excellent yield (86%) was obtained for the synthesis of a precursor of schindilactone A (**189**) containing a [5-7-8-5] ring structure.

### 6 Lewis-acid-promoted cyclization

Several Lewis acid-promoted intramolecular cyclization reactions of appropriate substrates were proposed to access the [5-8] bicyclic scaffold including the Nazarov cyclization, the Nicholas reaction or a Mukaiyama-type aldolization.

#### 6.1 Synthesis of fusicoauritone: Nazarov cyclization

**Scheme 38:**
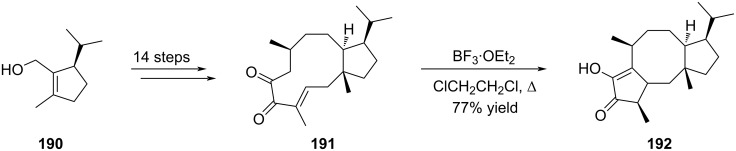
Nazarov cyclization revealing the fusicoauritone core structure **192**.

In the case of fusicoauritone (**28**), Williams envisaged a Nazarov cyclization to construct the bicyclo[3.6.0]undecane ring system in one step from an advanced intermediate [84]. The strategy proposed here was inspired by biogenetic considerations. Indeed, intermediate **191** possessing an eleven-membered ring could be considered as an equivalent of 3,7-dolabelladiene, the key biogenetic precursor of fusicoccanes, neodolabellanes as well as clavulananes. Following this hypothesis, exposure of compound **191**, prepared in 11 steps from cyclopentenol **190**, to BF_3_·OEt_2_ induced an intramolecular five-membered ring formation revealing the central cyclooctane ring of fusicoauritone (**192**) in 77% yield (Scheme 38).

**Scheme 39:**
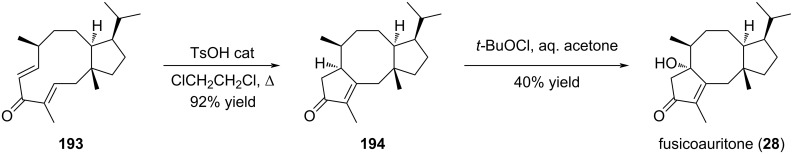
Synthesis of fusicoauritone (**28**) through Nazarov cyclization.

This approach was further extended to compound **193** which was cyclized in the presence of *p*-toluenesulfonic acid to furnish the corresponding tricyclic compound **194** in 92% yield. The latter was finally converted to fusicoauritone (**28**) upon direct oxidative treatment with *tert*-butyl hypochlorite in 40% yield (Scheme 39).

#### 6.2 Synthesis of epoxydictymene (**5**): Nicholas reaction

**Scheme 40:**
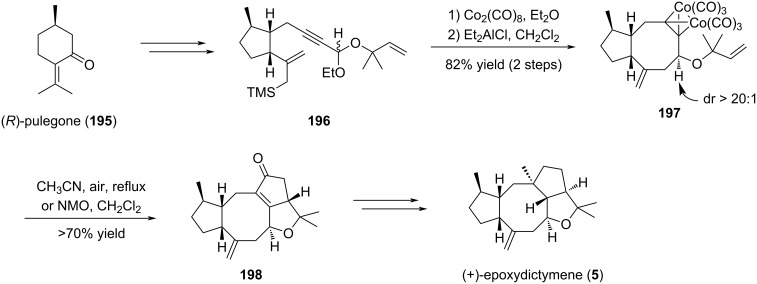
(+)-Epoxydictymene (**5**) synthesis through a Nicholas cyclization followed by a Pauson–Khand reaction to build the overall tetracyclic backbone.

In the synthesis of (+)-epoxydictymene (**5**), Schreiber proposed an elegant sequence involving a Nicholas cyclization followed by a Pauson–Khand reaction to build the overall tetracyclic backbone [85–86]. The Nicholas reaction took place on allylsilane **196** prepared from (*R*)-pulegone (**195**). After complexation of the triple bond with dicobalt octacarbonyl, the resulting red organometallic cluster underwent a cyclization in the presence of Et_2_AlCl to give the fused [5-8] ring system **197** in 82% yield over two steps and a high degree of stereoselectivity. Compound **197** was then engaged in a Pauson–Khand reaction to finalize the construction of the sesquiterpene core which ultimately lead to epoxydictymene (**5**) (Scheme 40).

#### 6.3 Synthesis of aquatolide: Mukaiyama-type aldolisation

**Scheme 41:**
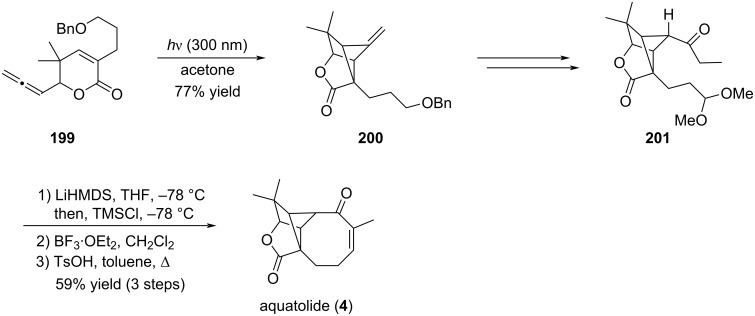
Synthesis of aquatolide (**4**) by a Mukaiyama-type aldolisation.

The synthetic plan reported by Hiemstra to synthesize aquatolide (**4**) takes advantage of their previous work on a photochemical [2 + 2]-cycloaddition to access the crucial bicyclo[2.2.1] hexane core and a late-stage intramolecular Mukaiyama-type aldol reaction to form the eight-membered ring [87]. To this end, ketone **201** was regioselectively converted to the enol silyl ether and directly subjected to cyclization upon exposure to BF_3_·OEt_2_ to give a mixture of stereoisomers. Final treatment with TsOH in refluxing toluene afforded aquatolide (**4**) as a crystalline product in 59% yield over the three step sequence (Scheme 41).

### 7 Rearrangement: synthesis of variecolin (**3**) with tandem Wolff/Cope rearrangement

**Scheme 42:**
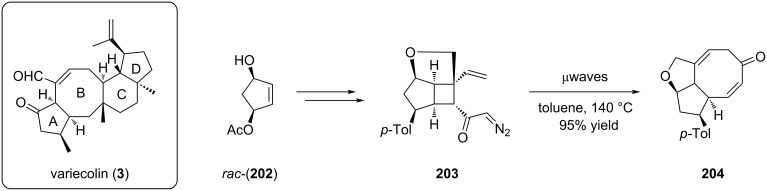
Tandem Wolff/Cope rearrangement furnishing the A-B bicyclic moiety **204** of variecolin.

Stoltz reported a stereoselective access to the bicyclo[3.6.0]undecane core through a tandem Wolff/Cope rearrangement approach [88]. The strategy was first designed in a racemic version and further developed in an asymmetric fashion. The prerequisite for the rearrangement was the presence of a diazocyclobutyl ketone as represented by compound **203**. When this strained bicycle was heated under microwave irradiation, the tandem Wolff/Cope rearrangement occurred to furnish the corresponding fused [5-8] carbocycle **204** in very high yield (Scheme 42).

**Scheme 43:**
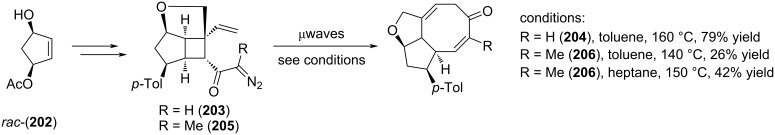
Asymmetric synthesis of the A-B bicyclic core **205** and **206** of variecolin.

Extension to the asymmetric version corresponding to the A-B ring of variecolin (**3**) demonstrated the importance of non-polar solvents for this tandem reaction. Thus, replacement of toluene by heptane improved the yield of the reaction in the presence of an additional methyl substituent (**206**, 26% to 42%) (Scheme 43).

### 8 Cycloaddition reactions

As exemplified above, access to fused bicycles with one cyclooctanoid ring suffers from major drawbacks including ring strains, disfavored enthalpy and entropy, and transannular hindrance. Among the available methods described in the literature, cycloaddition reactions constitute an efficient route that combines both one-pot cascade and good stereocontrol of the newly formed asymmetric centers. Three types of cycloadditions have recently been reported depending on the nature of the activation, thermal, metal-catalyzed, or photocatalyzed.

#### 8.1 Cycloadditions under thermal activation

**Scheme 44:**
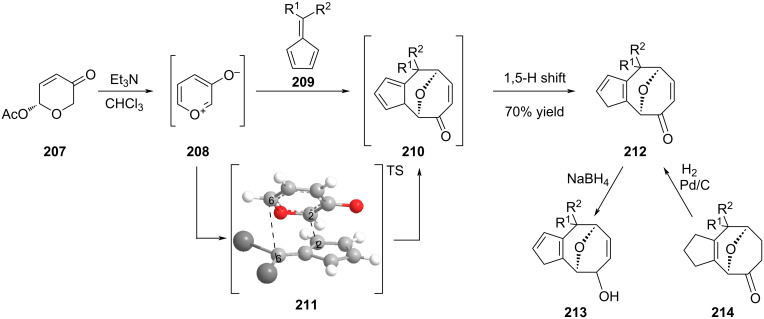
Formation of [5-8]-fused rings by cyclization under thermal activation.

Recently, an efficient synthesis of [5-8] fused oxabridged cyclooctanoids was reported by Radhakrishnan et al. [89]. They described the first [6 + 3] cycloaddition reaction between 6,6-diarylfulvene **209** and the betaine 3-oxidopyrylium (**208**) to give intermediate **210** that rearranged itself upon 1,5-hydrogen shift into **212** (Scheme 44). The reaction turned out to be regioselective and ab initio calculations rationalized a stereoselective *endo*-approach between the diene and the dienophile leading to the formation of C2_(fulvene)_–C2_(betaine)_ and C6_(fulvene)_–C6_(betaine)_ bonds [90]. The scope of the reaction was successfully extended to diaryl, dialkyl (including cycloalkyl), and aryl alkyl fulvenes. In the last examples, the stereogenicity of the C6 center of the newly formed cyclooctanoid ring was not controlled. Interestingly the [5-8]-fused ring scaffold possessed functions like an oxabridge, an α,β-unsaturated ketone and cyclopentadiene that can be derivatized at will [91]. For example, the dipolar cycloaddition on the olefin part of the enone allowed the synthesis of a [5-8-5] system while reduction gave access to either allylic alcohol **213** or ketone **214**.

**Scheme 45:**
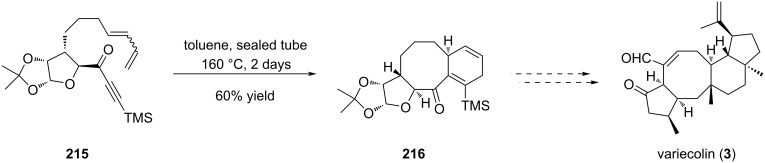
Construction of the [5-8-6] tricyclic core structure of variecolin (**3**) by Diels–Alder reaction.

Ghosh also reported a rapid access to the [5-8-6] ring system of variecolin (**3**) through an intramolecular Diels–Alder approach [92]. The key annulation step was achieved on compound **215** which was prepared from the carbohydrate chiral pool. The cycloaddition proceeded upon heating in a sealed tube over two days to give tricycle **216** in 60% yield (Scheme 45). The stereoselectivity of the reaction was controlled by the chiral pentoside unit. Interestingly, compound **216** possesses suitable function for further derivatization into variecolin (**3**). This study highlighted once again the potency of the Diels–Alder reaction in synthesis with the formation of a fused [6-8] ring system.

#### 8.2 Metal-catalyzed cycloadditions

**Scheme 46:**
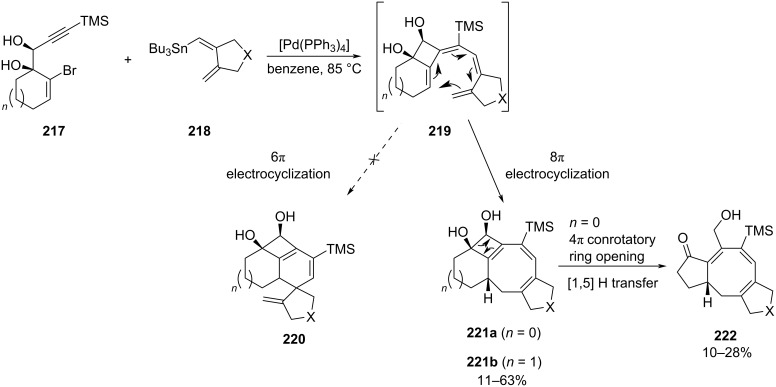
Synthesis of the [6-4-8-5]-tetracyclic skeleton by palladium-mediated cyclization.

The efficient use of transition-metal catalysis to perform the direct formation of a [5-8-5] tricyclic skeleton was described in the context of the ophiobolan core synthesis. Previous work highlighted the efficient cyclocarbopalladation of propargylic diol **217** in the presence of vinylstannane **218** followed by a 6π electrocyclization to give the unusual tricyclic system [93]. Using stannylated dienes **218**, the same cascade involving 4-*exo*-*dig* cyclization followed by a Stille coupling provided tetraene intermediates **219** (Scheme 46) [94]. In this case the 6π electrocyclization was unfavored as the resulting spirocyclic diene **220** was too strained. Instead, an 8π electrocyclization occurred to lead to the corresponding tetracyclic triene **221** as a pure diastereomer. Overheating of the reaction media caused the 4π electrocyclic ring opening of **221a** followed by a 1,5-hydrogen shift to provide ketoallylic alcohol **222**. Interestingly, using six-membered ring diols allowed to efficiently access in one pot the stable [6-4-8-5]-tetracyclic system **221b**.

**Scheme 47:**
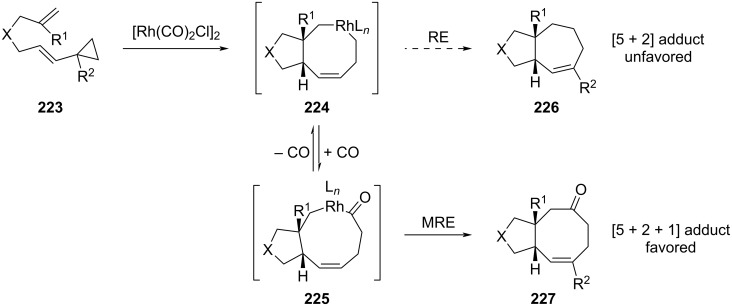
Access to the [5-8] bicyclic core structure of asteriscanolide (**227**) through rhodium-catalyzed cyclization.

Another metal-catalyzed cycloaddition was also reported in the course of the synthesis of the asteriscan family. The first total synthesis of the asteriscanolide (**2**) intricate natural product was described by Wender and co-workers in the late 90’s [95]. They employed a Ni(0)-catalyzed intramolecular [4 + 4] cycloaddition as the key step toward the tricyclic core. Later on, Yu et al. reported the one-pot rhodium(I)-catalyzed [(5 + 2) + 1] cycloaddition of a tethered ene-vinylcyclopropane in the presence of CO [96]. They argued that while reductive elimination (RE) of the rhodium intermediate **224** was unfavored, the introduction of a CO unit in the catalytic step followed by migratory reductive elimination (MRE) would be easier (Scheme 47). Indeed, the cycloaddition of the ene-vinyl cyclopropane **223** in the presence of [Rh(CO)_2_Cl]_2_ and 1 atm of CO gave cycloadduct **227** as a single diastereomer with more than 70% yield. Interestingly, this reaction was tolerant to geminal function as well as heteroatoms. Also, the geometry of the double bond in **223** influenced the *cis*–*trans* ratio of the final products.

**Scheme 48:**
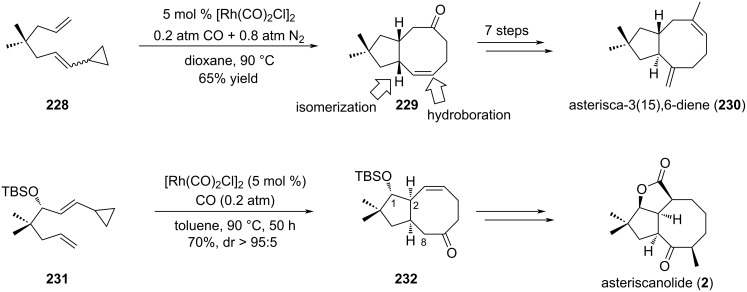
Total syntheses of asterisca-3(15),6-diene (**230**) and asteriscanolide (**2**) with a Rh-catalyzed cyclization as the key step.

This powerful strategy was then applied to the stereoselective total synthesis of (±)-asterisca-3(15),6-diene (**230**) and (+)-asteriscanolide (**2**). The simpler [5-6] bicycle was obtained from bicyclic *cis*-cyclooctenone **229** that arose from a previous [(5 + 2) + 1] cycloaddition of ene-vinylcyclopropane **228** (Scheme 48) [97]. A hydroxy group was then introduced thanks to a hydroboration reaction. Isomerization of position C7 was performed in acidic conditions to provide the targeted compound **230** with 14% overall yield over seven steps. The unusual [6.3.0] carbocyclic system bridged by a butyrolactone was the object of a more complex strategy. Selection of the appropriate ene-vinylcyclopropane substrate led to extensive work which ultimately allowed to consider enantiopure precursor **231** for the rhodium(I)-catalyzed cycloaddition [98]. Thus, exposure of **231** to [Rh(CO)_2_Cl]_2_ complex in toluene under controlled CO/N_2_ atmosphere yielded cyclooctenone **232** in 70% yield and high diastereoselectivity (>95:5). The presence of the TBS protecting group was critical for the stereoselectivity and the use of toluene rather than dioxane as initially described allowed to improve the yield from 30% to 70%. Moreover, the *cis* ring junction was in accordance with the configuration of the natural product. Density functional theory calculations were also undertaken to support the observed diastereoselectivity (Scheme 48) [40]. Interestingly, none of the other envisaged ene-vinylcyclopropanes were able to cyclize, highlighting the fact that preexisting five-membered rings were not suitable for this [(5 + 2) + 1] cycloaddition. Having intermediate **232** in hands, further transformations were performed: introduction of a hydroxy group at C8 following a 3-step procedure (enolization, iron-catalyzed cross-coupling and epoxidation); inversion of the configuration at C1 by an oxidation–reduction sequence and construction of the butyrolactone ring by free radical annulation of seleno carbonate.

#### 8.3 Photocyclizations

**Scheme 49:**
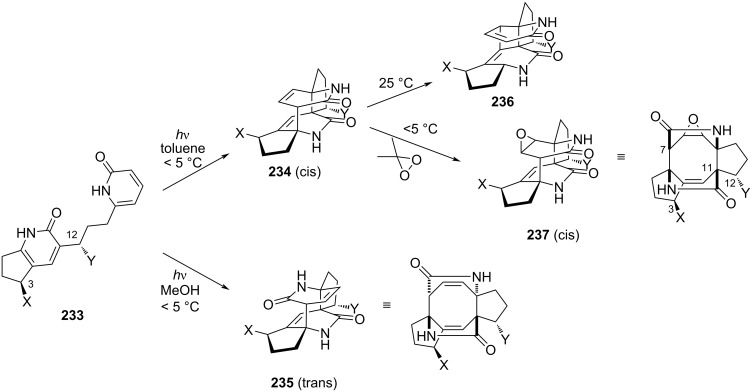
Photocyclization of 2-pyridones to access the [5-8-5] backbone of fusicoccanes.

Historically, the first attempt to attain the fusicoccan ring thanks to photocyclization started from linked 2-pyridones **233** (Scheme 49). Under irradiation provided by a 450 W mercury lamp, smooth conversion to dicyclopenta[*a*,*d*]cyclooctane was observed [99]. The intramolecular [4 + 4] cycloaddition allowed to control the stereoselectivity of C11 under the influence of C3 and C12. As for C7, the possibility to form H-bonds with the solvent greatly influences its stereogenicity. In the absence of *N*-methyl groups, polar solvents where strong H-bond networks exist lead to the *trans*-diastereomer **235** mainly, while non-polar solvents (like benzene) exclusively gave the *cis* isomer **234** [100]. This last compound quickly underwent irreversible Cope rearrangement at room temperature. To avoid this side reaction and to provide the targeted cyclooctane ring, a subsequent epoxidation was performed to give the corresponding epoxide **237**. Remarkably, such epoxidation reaction is both facial and site selective. Finally, selective reductive opening of one lactam ring was performed after activation as urea to provide the corresponding carbinol.

**Scheme 50:**
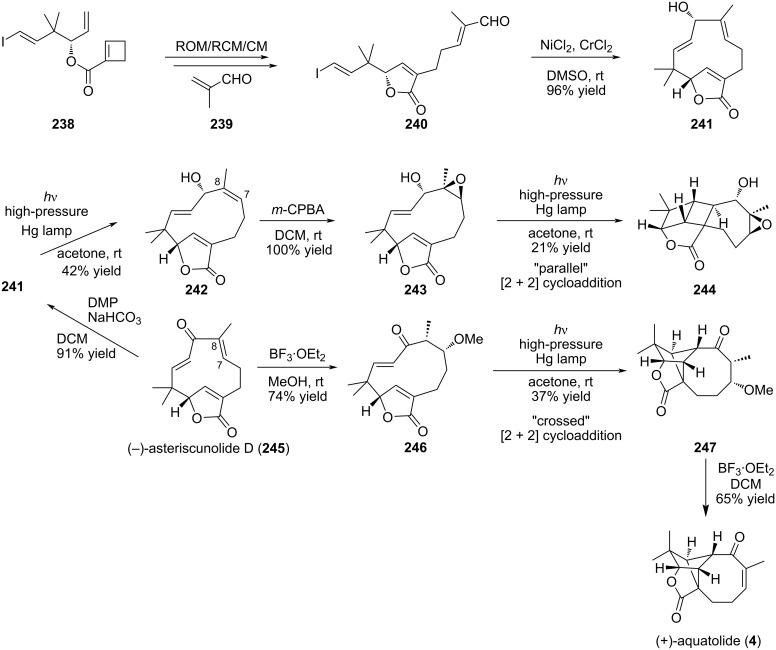
Total synthesis of (+)-asteriscunolide D (**245**) and (+)-aquatolide (**4**) through photocyclization.

Another straightforward access to the [5-8-5]-fused tricycle of the humulane core (see aquatolide (**4**)) has been reported using light-assisted cycloaddition. The humulane core was synthesized according to different methods including Mukaiyama aldol reaction [87] or intramolecular NHK strategy [62]. Recently, an elegant transannular [2 + 2] “crossed” cycloaddition was described by the group of Takao to obtain selectively (+)-aquatolide (**4**) from the asteriscanolide-type precursor **245** (Scheme 50) [101]. This is one of the first examples of a biomimetic transannulation strategy. It relied on the pioneering work of Li and co-workers on the isomerization of eleven-membered ring humulanes under irradiation [102]. In the report from Takao et al., the [2 + 2] photocycloaddition was performed starting from the same humulane ring using a high pressure mercury lamp (100 W) with a wide range of wavelengths to give the [5-5-4-8] ring system with moderate yield. The eleven-membered ring precursor **241** was obtained thanks to a ring-opening/ring-closing/cross-metathesis (ROM/RCM/CM) cascade followed by intramolecular Nozaki–Kishi reaction starting from the cyclobutenecarboxylate **238**. Remarkably, compound **241** was obtained as a single diastereomer owing to the fact that the pseudoequatorial hydroxy group is favored in the NHTK reaction [103]. Interestingly, the intermediate, humulene lactone **241**, is common to related natural products. Success of the crucial [2 + 2] cycloaddition was obtained after masking of the double bond C7–C8 to prevent simple isomerization. Transformation into an epoxide lead to the alternative parallel cycloaddition to give the regioisomer of aquatolide **244** but after masking the olefin as methyl ether the bicyclo[2.1.1]hexane **247** was obtained with 37% yield. Elimination of methanol under acidic conditions gave the corresponding aquatolide (**4**). It confirms the crucial role of the alcohol or thiol function at position C6 in the natural precursor to provide the aquatolide skeleton by the plant. Conformational analyses confirmed the preferred cross-path adopted by intermediate **246**. It is in accordance with the fact that intramolecular [2 + 2] photocycloadditions prefer to form five-membered ring regioisomers [104].

### 9 Miscellaneous

#### 9.1 Biocatalysis

**Scheme 51:**
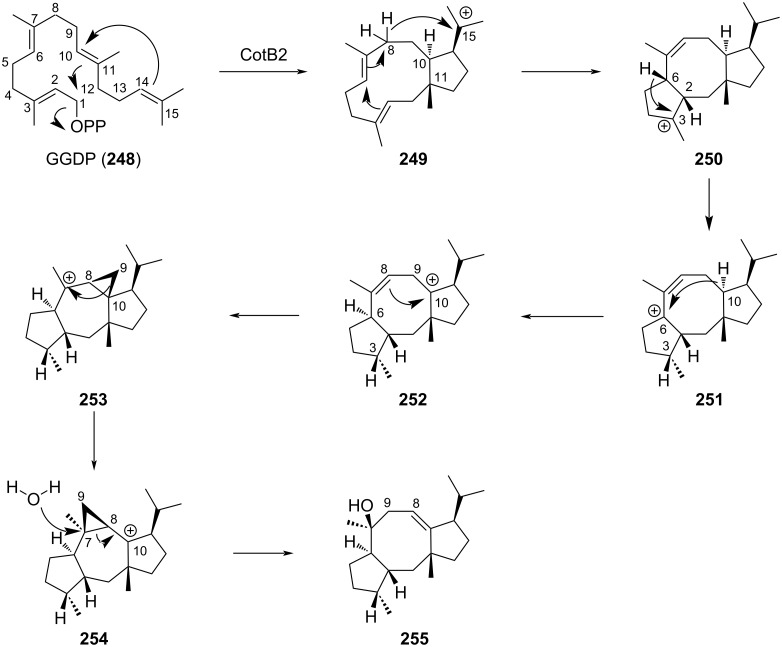
Biocatalysis pathway to construct the [5-8-5] tricyclic scaffold of brassicicenes.

Despite cutting-edge organic methods available nowadays for the production of fused bicycle cyclooctatin, numerous drawbacks remain including lengthy synthetic pathways, adequate control of the stereochemistry and low yields. The diversity of enzymes involved in the biosynthetic pathway could constitute an alternative to a total synthesis approach. Significant advantages of such a process are the high selectivity of the enzyme towards the substrate and the clean conversion to one and only one product. Thus, it reduces drastically the number of steps involved, the amount of waste generated and the need for purification. As many enzymes work in aqueous media under mild conditions, often at room temperature, the fingerprint on the environment is low and the amount of energy used is minimal. Biosynthetic pathways toward terpene cyclization are now well documented [105–106] but only two terpene cyclases involved in the [5-8-5]-fused ring structure have been reported so far [107–108]. They both used geranylgeranyl diphosphate (GGDP **248**; C20) as precursor. Recently, the group of Oikawa managed to rebuild the biosynthetic pathway of brassicicenes thanks to the heterologous expression of eight genes including a *N*-terminal terpene cyclase BscA [109]. Earlier on, Kuzuyama et al. deciphered the mechanism involved in the biosynthesis of cyclooctat-9-en-7-ol **255** thanks to the combination of theoretical calculations, in vivo studies using ^13^C-labeled glucose and in vitro reaction with deuterated GGDP [110–111]. They showed that the reaction cascade catalyzed by the cyclooctat-9-en-7-ol synthase CotB2, a class I terpene cyclase derived from *Streptomyces*, was divided in 3 parts (Scheme 51); i) the ring construction that involved the formation of carbocation and subsequent electrophilic cyclization to provide the bicyclic cationic intermediate **249**. Hydride-shift cascade gave the core tricyclic [5-8-5] fused ring structure **250**, ii) long-range cation transfer via multiple hydride migration to generate carbocation **252**, and iii) carbon–carbon bond rearrangement through the formation of cyclopropylcarbinyl cations **253** and **254**. Finally, attack of water on the cyclopropyl gave the targeted monounsaturated cyclooctenol **255**.

**Scheme 52:**
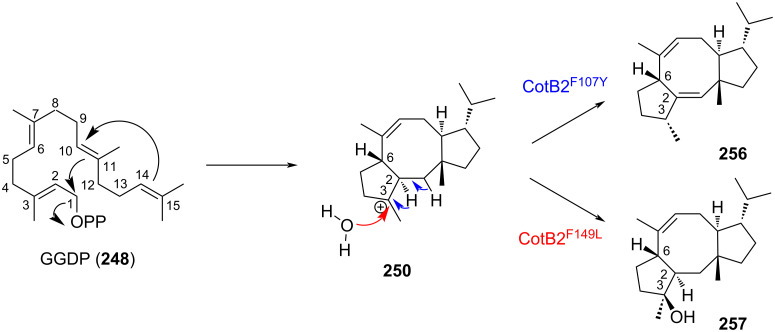
Influence of the CotB2 mutant over the cyclization’s outcome of GGDP.

Remarkably, conformation of the starting GGDP (**248**) within the catalytic pocket as well as the cyclization mechanism described succinctly above allowed the strict control of the 6 stereocenters of **255** starting from the achiral C_20_ allylic diphosphate GGDP (**248**). This specificity for one single product was further diverted for the biocatalyzed synthesis of non-natural analogs of **255**. Site-saturation mutagenesis of CotB2 allowed to identify two mutants that catalyzed the conversion of GGDP into hitherto unknown fusicoccane macrocycles **256** and **257** (Scheme 52) [112]. In both mutants, swapping the hydrophobic amino acids phenylalanine 149 and 107, involved in the catalytic pocket, with shorter leucine and alanine, respectively modified either the spatial constraints or the charge profile of the active pocket.

To conclude this part, biocatalysis using mutated versions of cyclooctanol synthase constitutes an elegant and efficient alternative to classical multistep synthesis. The cost associated to GGDP (**248**) is now lifted thanks to the development of alternative production systems and it therefore paves the way to an innovative eco-friendly synthesis of [5-8-5] fusicoccane scaffolds.

## Conclusion

The [5-8] bicyclic motif encountered in the sesquiterpene, diterpene, and sesterterpene series has stimulated the chemistry community to develop efficient and straightforward synthetic methods, the main challenge consisting in the construction of the eight-membered ring. The intramolecular ring-closing metathesis (RCM) has attracted considerable attention due to its versatility, functional group tolerance and mild reaction conditions and represents the method of choice to access this scaffold. Indeed, two strategies have been designed to construct the eight-membered ring: early- or late-stage cyclization. The Grubbs II (G-II) catalyst appears to be the most versatile catalyst for this transformation. However, Grubbs I (G-I) and Hoveyda–Grubbs II (HG-II) catalysts were also used in some situations. Of particular interest, the tandem ring closing metathesis, allowing the formation of the [5-8] motif in a single step, constitutes a very powerful method. Among the most recent approaches described, the intramolecular radical cyclization proves to be very attractive and several groups focused on this strategy. Interestingly, radical-promoted intramolecular eight-membered ring-closing methodologies were successfully applied on advanced intermediates, delivering the cyclic compounds in high yields and high stereoselectivity. Thus, elaboration of pleuromutilin (**1**) and several analogs constituted a representative example showcasing the usefulness of this strategy. Also, cycloaddition reactions, either under thermal activation, metal-promoted or under photoactivation have been explored. However, applications are mainly confined to the preparation of a [5-8] bicyclic model mimicking the natural product. Finally, exploration of the biogenetic pathways allowed to propose some elegant biocatalytic approaches which pave the way to innovative eco-friendly syntheses.

## Appendix

Abbreviations used in text, figures and schemes are collected in Table 1.

**Table 1:** List of abbreviations.

Ac	acetate
acac	acetylacetone
aq	aqueous
BEt_3_	triethylborane
Bn	benzyl
Boc	*tert*-butyloxycarbonyl
Bz	benzoate
CAN	ceric ammonium nitrate
Cat	catalytic
CM	cross metathesis
cod	cyclooctadiene
Cy	cyclohexane
DA	Diels–Alder
DCM	dichloromethane
DDQ	2,3-dichloro-5,6-dicyano-1,4-benzoquinone
DFT	density functional theory
DLP	dilauroyl peroxide
DMF	dimethylformamide
DMSO	dimethyl sulfoxide
DMP	Dess–Martin periodinane
dppf	diphenylphosphinoferrocene
dppp	bis(diphenylphosphino)propane
dr	diastereoisomeric ratio
E	electrophile
Et	ethyl
Et_2_O	diethyl ether
Et_3_N	triethylamine
EtOAc	ethyl acetate
EYRCM	enyne ring-closing metathesis
GGDP	geranylgeranyl diphosphate
HIV1	Human Immunodeficiency Virus 1
HMPA	hexamethylphosphoramide
iPr	isopropyl
LED	light-emitting diode
*m*-CPBA	*m*-chloroperoxybenzoic acid
M	mol/L
Me	methyl
MeOH	methanol
MOM	methoxymethyl
MRE	migratory reductive elimination
NHC	N-heterocyclic carbene
NHK	Nozaki–Hiyama–Kishi
NMO	*N*-methylmorpholine *N*-oxide
Ph	phenyl
PhH	benzene
PhOK	potassium phenolate
Piv	pivaloyl
PMB	*para*-methoxybenzyl
PP	pyrophosphate
*p*-Tol	*p*-tolyl
quant	quantitative
Rac	racemic
RCM	ring-closing metathesis
RE	reductive elimination
ROM	ring-opening metathesis
rt	room temperature
SAR	structure–activity relationship
SE	electrophilic substitution
SEM	trimethylsilylethoxymethyl
TADDOL	α,α,α’,α’-tetraaryl-2,2-disubstituted 1,3-dioxolane-4,5-dimethanol
TBAF	tetrabutylammonium fluoride
TBDPS	*tert*-butyldiphenylsilyl
TBS	*tert*-butyldimethylsilyl
*t*-Bu	*tert*-butyl
*t*-BuOH	*tert*-butanol
*t*-BuLi	*tert*-butyllithium
TEMPO	(2,2,6,6-tetramethylpiperidin-1-oxyl
TES	triethylsilyl
Tf	triflate
THF	tetrahydrofuran
TIPS	triisopropylsilyl ether
TMS	trimethylsilyl
TMSCl	trimethylsilyl chloride
TRCM	tandem ring closing metathesis
Ts	tosyl
TsOH	*p*-toluenesulfonic acid
UV	ultraviolet
W	Watt
